# Penetratin Decoration Increases RNAi Silencing Effects of Polymeric Carriers

**DOI:** 10.1021/acs.chemmater.6c00522

**Published:** 2026-04-27

**Authors:** Salvatore Emanuele Drago, Marta Cabibbo, Cinzia Scialabba, Emanuela Fabiola Craparo, Gennara Cavallaro

**Affiliations:** Lab of Biocompatible Polymers, Department of Biological, Chemical and Pharmaceutical Sciences and Technologies (STEBICEF), 18998 University of Palermo, Via Archirafi 32, Palermo 90123, Italy

## Abstract

This study reports the development of a polymeric carrier for pulmonary siRNA delivery via inhalation. Poly­(α,β-N-(2-hydroxyethyl)-d,l-asparamide) (PHEA) was first functionalized with divinyl sulfone (DV) to form PHEA-VS, enabling controlled grafting of 1,2-bis­(3-aminopropylamino)­ethane (bAPAE, of about ∼25 mol %) and poly­(2-methyl-2-oxazoline) (PMeOx, of about ∼5 mol %). The resulting PHEA-VS-*g*-(PMeOx;bAPAE) copolymer contained protonable amines for efficient siRNA complexation. Potentiometric titration confirmed strong buffering capacity, while fluorescence studies indicated pH-responsive membrane interaction, suggesting improved endosomal escape. Polyplexes formed starting from a polymer/siRNA ratio of 5, with diameters below 40 nm, showing stability in mucins and pulmonary surfactant and protection against RNase degradation. Surface decoration with the cell-penetrating peptide penetratin (Pen) via a terminal alkyne on PMeOx enhanced cellular uptake and siRNA release. Biocompatibility tests on 16-HBE cells showed viability over 80% at high polymer concentrations. Functional assays in MDA-MB-231-LUC cells demonstrated effective gene silencing, particularly at a polymer/siRNA ratio of 5. Combined with the favorable aerosolization properties of aqueous dispersions, these results highlight PHEA-VS-*g*-(PMeOx;bAPAE) as a versatile platform for pulmonary siRNA delivery, offering stability, biocompatibility, and targeted intracellular release for potential treatment of respiratory diseases.

## Graphical abstract

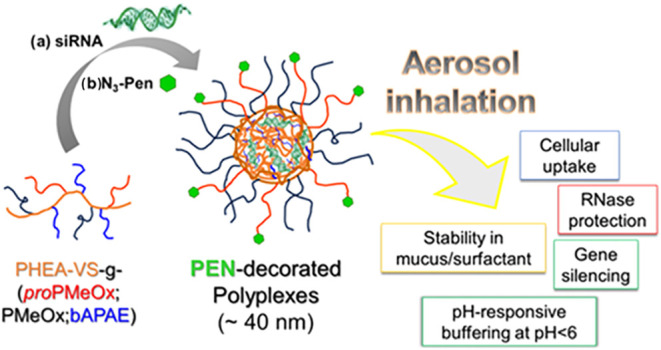

## 1 Introduction

RNA interference (RNAi), mediated by small interfering RNA (siRNA), provides a specific mechanism for gene silencing and in principle is promising as a therapeutic modality for chronic pulmonary diseases like chronic obstructive pulmonary disease (COPD), cystic fibrosis (CF), and severe asthma.^1^ The inhalation route enables direct delivery to the pulmonary epithelium, maximizing local efficacy while minimizing systemic exposure and degradation. Nonetheless, successful pulmonary siRNA therapy is challenged by multiple physiological barriers, including the viscoelastic mucus gel, lipid-rich surfactant, events that hinder reaching airway epithelial cells where siRNA should exert their effects.^2–4^

To overcome these hurdles, research has focused on engineering nanoplatforms able to protect siRNA from degradation, facilitate transport through mucus and surfactant layers, and enhance cellular uptake while promoting endosomal escape.^5^ Among such strategies, polymer-based polyplexes stand out for their structural tunability, biocompatibility, and capacity to condense nucleic acids into stable nanoscale complexes^6,7^ siRNA carriers are typically cationic polymers, which bind nucleic acids via electrostatic interactions. Polyethylenimine (PEI) has long served as a benchmark for nonviral gene delivery due to its high transfection efficiency; however, its clinical use is limited by significant cytotoxicity and low biodegradability, challenges that are particularly critical in the pulmonary setting.^8^

To minimize undesired interactions with mucus and surfactant, pulmonary delivery carriers are often modified with hydrophilic polymers, with poly­(ethylene glycol) (PEG), traditionally considered as the gold standard.^9–11^ However, poly­(2-methyl-2-oxazoline) (PMeOx) has recently gained attention as a promising alternative, offering similar stealth properties while exhibiting lower immunogenicity and faster clearance.^12–14^ Its ability to reduce nonspecific adsorption and protein fouling makes PMeOx particularly advantageous for inhalable formulations, enhancing nanoparticle diffusion through mucus and facilitating more effective access to the airway epithelium.^15,16^

Beyond passive surface shielding, cell-penetrating peptides (CPPs), such as Penetratin (Pen),^17^ have emerged as powerful tools to enhance cellular uptake and transfection efficiency of siRNA. When properly exposed on the nanoparticle surface, CPPs can facilitate both cell internalization and endosomal escape, key steps for effective RNAi.^18–21^ It was already demonstrated that both cellular uptake and gene transfection increase with CPP surface coverage up to a plateau, indicating the presence of optimal density “windows” rather than a linear relationship.^22,23^ In contrast, excessive CPP densities and larger NP sizes are associated with increased lysosomal trapping and cytotoxicity, ultimately compromising delivery efficiency.^24,25^ Moreover, Khalil showed that carriers with near-neutral or slightly negative surface charge, while maintaining sufficient functional exposure of Pen, have shown improved performance, particularly in brain delivery contexts.^26^ Therefore, an appropriate carrier design, balance between CPP exposure and overall surface charge is therefore critical, and engineering strategies such as PEG spacers further enable fine control over CPP density and spatial presentation, reducing nonspecific interactions and toxicity while preserving translocation efficiency.^27^

Thanks to its biocompatibility and tunable functionability, poly­(α,β-*N*-(2-hydroxyethyl)-d,l-aspartamide) (PHEA) can be considered a very proper candidate to obtain cationic polymers able to give efficient polyplexes for siRNA delivery. Spermine or 1,2-bis­(3-aminopropylamino)­ethane (bAPAE) based cationic PHEA derivatives have already demonstrated excellent siRNA complexing ability, low cytotoxicity, and the capacity to transfect bronchial epithelial cells. Moreover, the polymer’s chemical high versatility for functionalization allowed the introduction of stealth groups and targeting ligands.^9,28,29^

In this context, a synthetic and versatile strategy to obtain a cationic multifunctional polymeric derivative has been designed and tested. A cationic and stealth PHEA derivative was synthesized by a divinyl sulfone-activated intermediate (PHEA-VS), which was subsequently grafted with bAPAE and PMeOx in a one-step process, obtaining the PHEA-VS-*g*-(PMeOx;bAPAE) graft copolymer. The ability of PHEA-VS-*g*-(PMeOx;bAPAE) to efficiently complex siRNA was confirmed at low polymer/siRNA weight ratio. To improve cellular uptake, the obtained PHEA-VS-*g*-(PMeOx;bAPAE)/siRNA polyplexes were functionalized on the surface with Pen by exploiting the terminal alkyne groups on the PMeOx chains and azide of Pen.

Finally, as a proof of concept, an aqueous dispersion of polyplexes was nebulized to generate an aerosol, and its aerodynamic properties were characterized to assess the potential for deep lung deposition.

## 2 Result and Discussion

### 2.1 Synthesis and Characterization of PHEA-VS-*g*-(PMeOx;bAPAE) Copolymer

Given the growing need for siRNA carriers suitable for efficient RNAi based therapy, synthetic polymers offer a versatile platform, as their structural and functional features can be precisely engineered to meet the specific siRNA requirements, such as protonability.^7^ This work describes the design of a polymeric carrier for siRNA delivery to the lung via inhalation, based on a synthetic graft copolymer of the α,β-poly­(*N*-2-hydroxyethyl)-d,l-aspartamide (PHEA). PHEA was selected as starting material because of it has already been successfully employed for efficient polymeric gene vectors,^9,28,29^ and other drug delivery systems.^30–34^ First, to ensure more controllable synthetic steps, PHEA underwent covalent modification through reaction with divinyl sulfone (DVS) (Scheme 1, step a). The degree of substitution of the resulting copolymer, named PHEA-VS, was determined to be 30.4 ± 1.4 mol % using ^1^H NMR spectroscopy (Figure S1, spectrum a), by comparing the vinyl proton signals (6.8, 6.4, and 6.3 ppm) to those of the PHEA repeating units (2.8 ppm). This process yielded a polymer capable of further finely tunable side-chain functionalization via Michael-type addition reactions with a variety of nucleophiles. To enable complexation of genetic material through electrostatic interactions and to minimize interaction with mucus components of the resulting carrier in the lung, the tetramine 1,2-bis­(3-aminopropylamino)­ethane (bAPAE) and the hydrophilic poly­(2-methyl-2-oxazoline) (PMeOx) were employed, respectively.^29,35^ PMeOx (−CH_3_ terminated) was synthesized by cationic ring-opening polymerization (CROP), under optimized conditions to obtain a product with an average molecular weight *M̅*_*w*_ of 5 kg/mol (Figure S2). The final copolymer, named PHEA-VS-*g*-(PMeOx;bAPAE), was obtained via a simply telescopic synthetic approach: initially, PHEA-VS was reacted with PMeOx in an aqueous environment (Scheme 1, step b), followed by the transfer of the reaction mixture into an organic solvent (DMF) containing an excess of bAPAE (Scheme 1, step c).

**Scheme 1:**
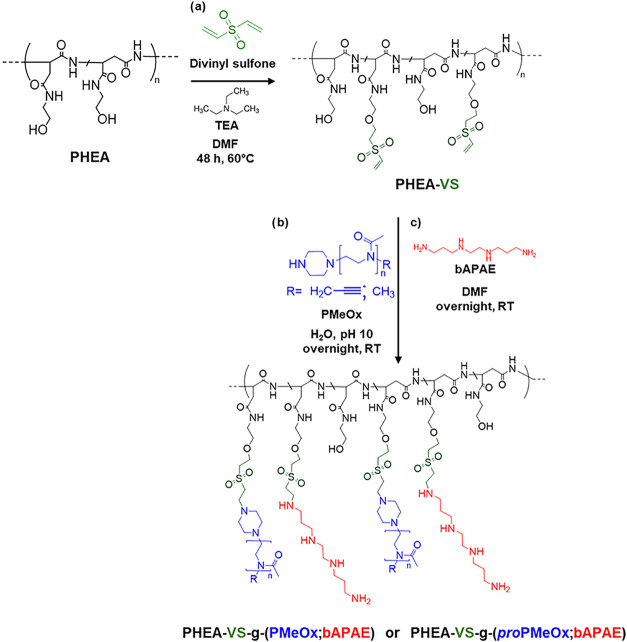
Synthetic Procedure to Obtain PHEA-VS (Step a), INU-VS-*g*-(PMeOx;bAPAE) or PHEA-VS-g-(*pro*PMeOx;bAPAE) (Step b and c)

The characterization of the obtained copolymer was carried out by recording two ^1^H NMR spectra due to the peak overlapping depending on the pH of the medium. The first was performed under acidic conditions (Figure S1, spectrum b) to determine the overall percentage of functionalized repeating units (RU) of PHEA backbone. This was achieved through comparison of the integral given by the CH_2_ protons of the total functionalized RU of PHEA (at δ 4.0 ppm) with that associated with CH_2_ protons of all RU (at δ 3.67 ppm). A second spectrum, reported in Figure 1, was recorded under basic conditions to quantify the extent of functionalization with PMeOx and bAPAE. Specifically, both the integrals given by the four protons of bAPAE (at δ 1.65 ppm), and those given by the 174 protons of PMeOx (at δ 2.1 ppm), were compared to the integral of the CH_2_ signal attributed to the PHEA backbone (δ 4.0 ppm), obtaining the degrees of derivatization (DD%) of 5.3 ± 1.1 mol % and 25.8 ± 1.4 mol %, respectively for PMeOx and bAPAE. Furthermore, the vinyl signals at 6.98, 6.52, and 6.41 ppm are absent, confirming complete functionalization of the VS groups in PHEA-VS.

**Figure 1:**
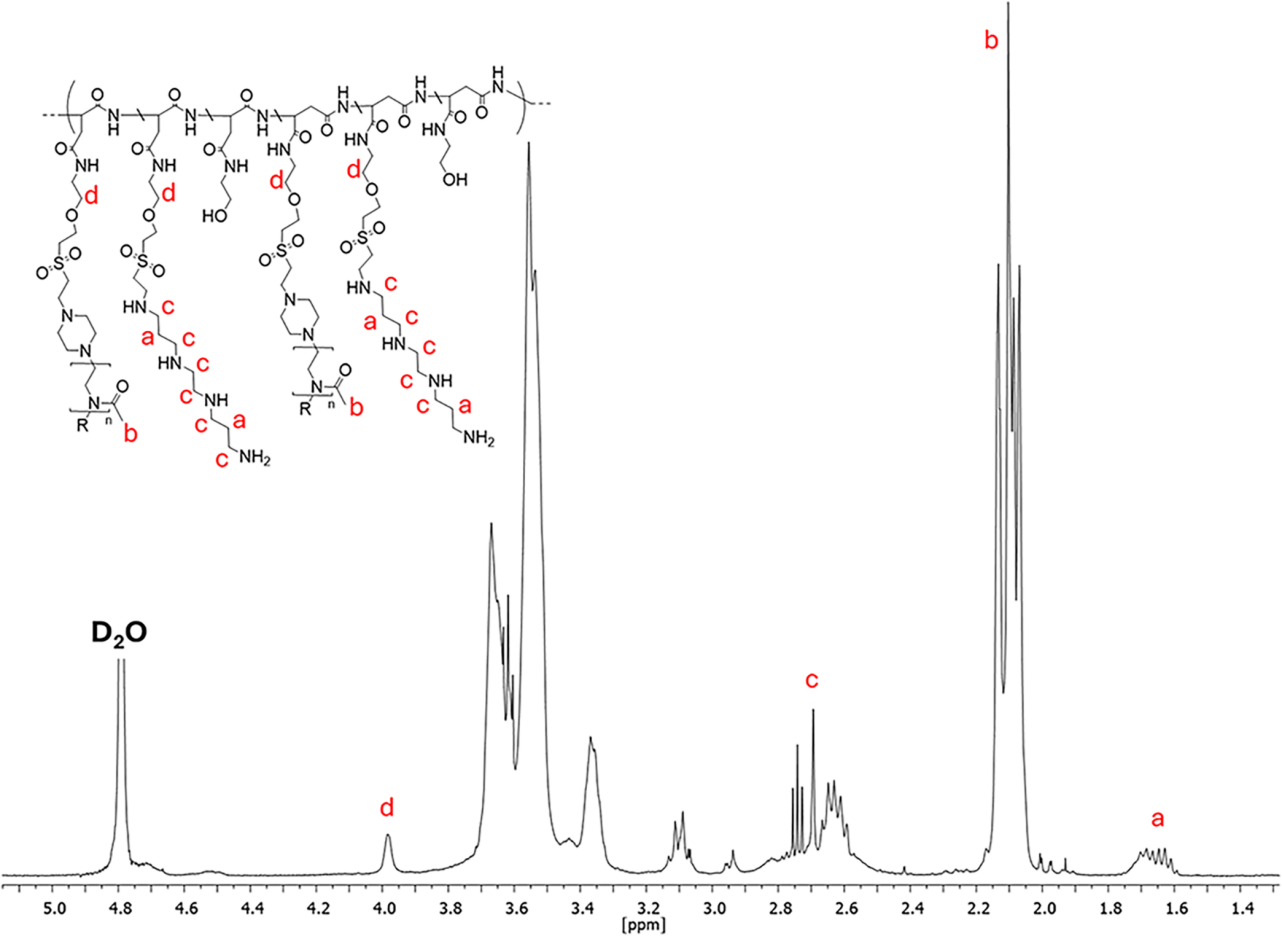
^1^H NMR spectra of PHEA-VS-*g*-(PMeOx;bAPAE) copolymer in D_2_O (pD ≈10).

The molecular weight distribution of both PHEA-*g*-VS and PHEA-VS-*g*-(PMeOx;bAPAE) copolymers was analyzed by size exclusion chromatography (SEC), and obtained data shown in Table 1. As it can be seen, the molecular weight of PHEA-VS-*g*-(PMeOx;bAPAE) derivative was lower than that of the starting polymer (PHEA-VS). This decrease can be attributed to the reaction conditions i.e., the use of a large excess of amine during the functionalization with bAPAE, which could induce partial cleavage of amide bonds in the polymer backbone through transamidation.

**Table 1:** Weight-Average Molecular Weight (*M̅_w_*), Weight-Average Molecular Numeric Weight (*M̅*_*n*_), Polydispersity Index (*M̅_w_*/*M̅_n_*), and Chemical Composition of Copolymers

	Molecular weight			Derivatization degree (DD%)		
Copolymers	*M̅_w_* (g/mol)	*M̅*_*n*_ (g/mol)	*M̅_w_*/*M̅_n_*	DD_VS_	DD_PMeOx_	DD_bAPAE_
PHEA	40100	54300	1.35	---	---	---
PHEA-VS	66700	49900	1.33	30.4 ± 1.4	---	---
PHEA-VS-*g*-(PMeOx;bAPAE)	55900	30400	1.84	--	5.3 ± 1.1	25.8 ± 1.4

To obtain a graft copolymer that could furtherly be modified with other ligands, the −C≡CH terminated PMeOx was also synthesized (Figure S3) and used in the telescopic synthesis reported in Scheme 1, obtaining the PHEA-VS-*g*-(*pro*PMeOx;bAPAE). PHEA-VS-*g*-(*pro*PMeOx;bAPAE) characterization by ^1^H NMR and SEC analyses showed that the derivative carrying −C≡CH terminated *pro*PMeOx chains is superimposable in terms of DD and *M̅_w_* values to that carrying −CH_3_ terminated PMeOx chains (data not showed). Therefore, conjugation with DV produces a copolymer that can be easily furtherly functionalized by using a one-pot process with different nucleophilic molecules.

The proton sponge effect in copolymers arises from the chemistry and spatial arrangement of amine groups, as well as from the type of amine, whose distributed protonation along the polymer chain provides effective buffering in the endosomal pH range (∼5–7).^36–38^ Moreover, by tuning p*K*_a_, amine density, distribution, and polymer architecture, copolymers can thus be designed for optimized proton sponge-mediated endosomal escape. To evaluate the buffering behavior of PHEA-VS-*g*-(PMeOx;bAPAE) in the pH range typical for proton sponge effect, due to the 25 mol % of RU carrying protonable amines, potentiometric titrations were performed (Figure 2a). In fact, these functionalities are critical for both the complexation of nucleic acids and for providing buffering capacity, which is key to promoting endosomal escape through the proton sponge effect.^39^ The results revealed that within the pH range of 7 to 5, critical for promoting endosomal escape through the proton sponge mechanism,^40^ the copolymer displayed a buffering capacity (β), defined as μmol HCl/ΔpH, of 8. This value is higher than that observed for bAPAE alone under identical conditions (β_bAPAE_ = 6). The enhanced buffering effect may be attributed to the conversion of primary amines into secondary amines upon their reaction with VS groups, which results in species with different p*K*_a_ values.^41^ Based on literature data,^42^ within the same pH range, PEI and PAMAM display β values of approximately 4.8 and 5.7 per mg of material, respectively. By comparison, the PHEA-VS-*g*-(PMeOx;bAPAE) graft polymer here described exhibits a β value of 0.26 per mg, roughly 20 times lower. This difference can be explained by considering that the copolymer is multifunctional, being made up of different components grafted onto the PHEA backbone: in fact, only about 9 wt % of the total weight corresponds to amine-containing units, while the other components (PHEA and PMeOx) are nonionic chains and do not contribute to the copolymer buffering capacity. To gain deeper insight into the protonation behavior across different pH levels, a reverse (backward) titration was also conducted (Figure 2b), and the data were analyzed using the De Levie method,^43^ applying the math equations reported in SI. Curve fitting analysis allowed the determination of the p*K*_a_ values associated with the amine groups in the polymer, which were found to be 9.93, 8.37, 6.24, and 4.26.

**Figure 2:**
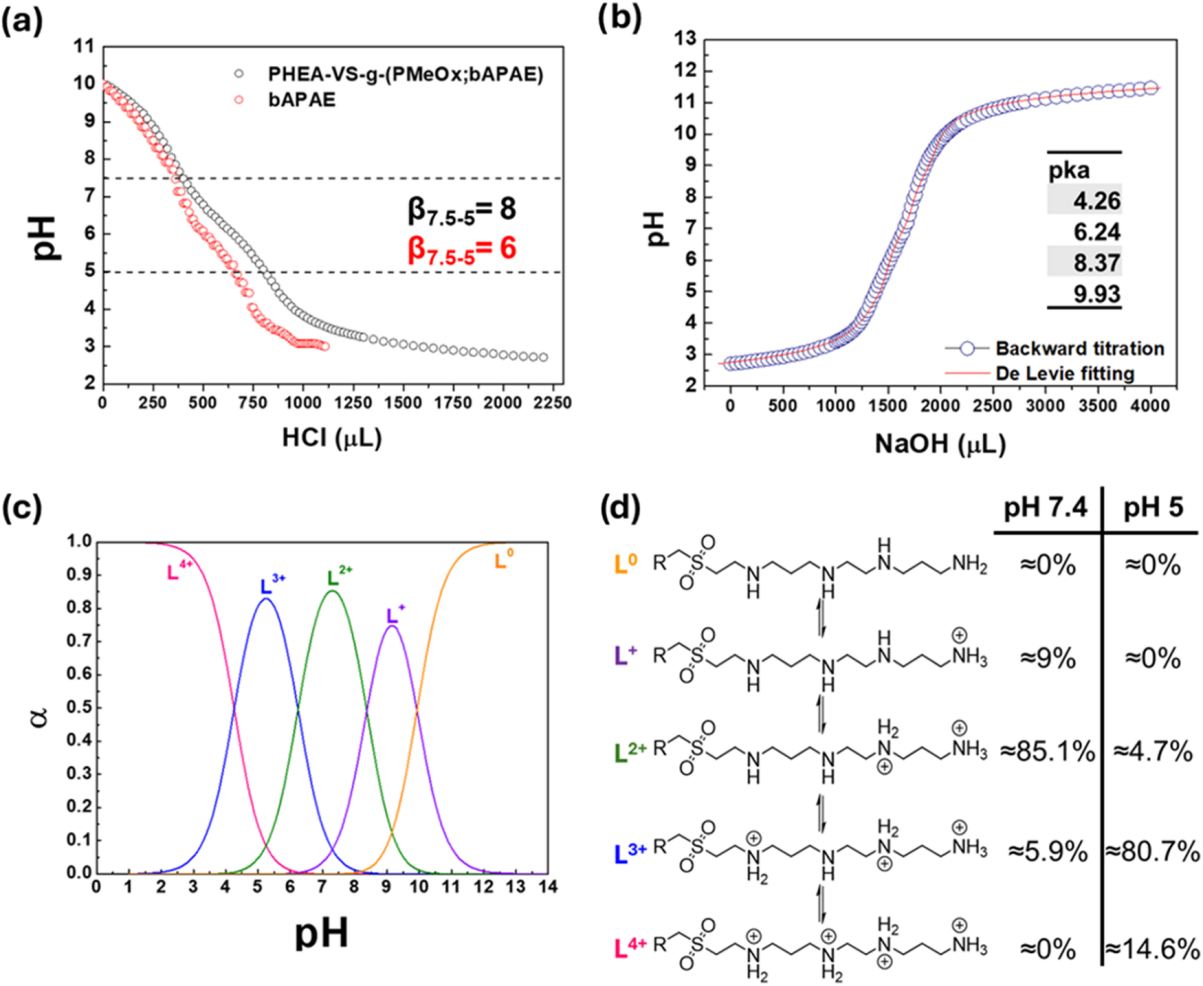
(A) Forward acid–base titration (NaOH volume versus pH) of PHEA-VS-*g*-(PMeOx;bAPAE) and bAPAE, (b) Backward acid–base titration (NaOH volume versus pH) of PHEA-VS-*g*-(PMeOx;bAPAE) and De Levie fitting curve, (c) speciation curves (α versus pH) for each α of PHEA-VS-*g*-(PMeOx;bAPAE) and (d) species abundance (%) at pH 7.4 and pH 5.

Based on these values, the corresponding speciation curves were generated (Figure[Fig fig2]c). As illustrated in Figure 2d, at pH 7.4, the copolymer exists predominantly in its diprotonated form (L^2+^), accounting for approximately 85% of the species, while the monoprotonated (L^+^) and triprotonated (L^3+^) forms contribute around 9% and 6%, respectively. Under more acidic conditions, the fraction of the fully protonated species (L^4+^) progressively increases, reaching about 15% at pH 5. At the same pH, the triprotonated species (L^3+^) became predominant, representing roughly 81% of the total population. This protonation behavior could be attributed to the retention of a secondary amine group for each functionalized RU, that is different from that showed by a PHEA-*g*-bAPAE derivative obtained by using a conjugation approach based on amide bond formation.^9^ In particular, at pH 7.4, the latter copolymer had a total protonation like the new one (70% L^2+^ and 30% L^+^), while at pH 5 the total protonation was lower (50% L^2+^ and 50% L^3+^).^9^ This difference highlights how the adopted synthetic strategy allows the modulation of the chemical properties of the resulting materials.

The different copolymer protonation as a function of pH could affect how it interacts with biological membranes, depending on the pH of the intracellular environment. The pH-responsive interaction may contribute to enhancing endosomal escape, offering an additional mechanism to support this critical step in intracellular delivery.^44,45^ To further investigate this aspect, an in vitro study was performed using human bronchial epithelial cells (16-HBE), employed here as a cell-based model to mimic endosomal/lysosomal membranes rather than the cytosolic membranes. In this experimental setup, cells were incubated with a PHEA-VS-*g*-(PMeOx;bAPAE) aqueous dispersion under controlled extracellular pH conditions designed to reproduce different intracellular compartments. Specifically, incubation at pH 7.4 was used as a reference condition, whereas incubation at pH 5.5 was intended to simulate the acidic luminal environment of endosomes/lysosomes, effectively treating the incubation medium as a proxy for the endosomal/lysosomal interior. Yellow Oxazole was employed as a fluorescent probe to evaluate membrane permeabilization. As shown in Figure 3, a pronounced intracellular fluorescence signal was detected only when cells were coincubated with the copolymer at pH 5.5, indicating enhanced membrane permeabilization under acidic conditions. Conversely, negligible intracellular fluorescence was observed at physiological pH or in buffer-only controls.

**Figure 3:**
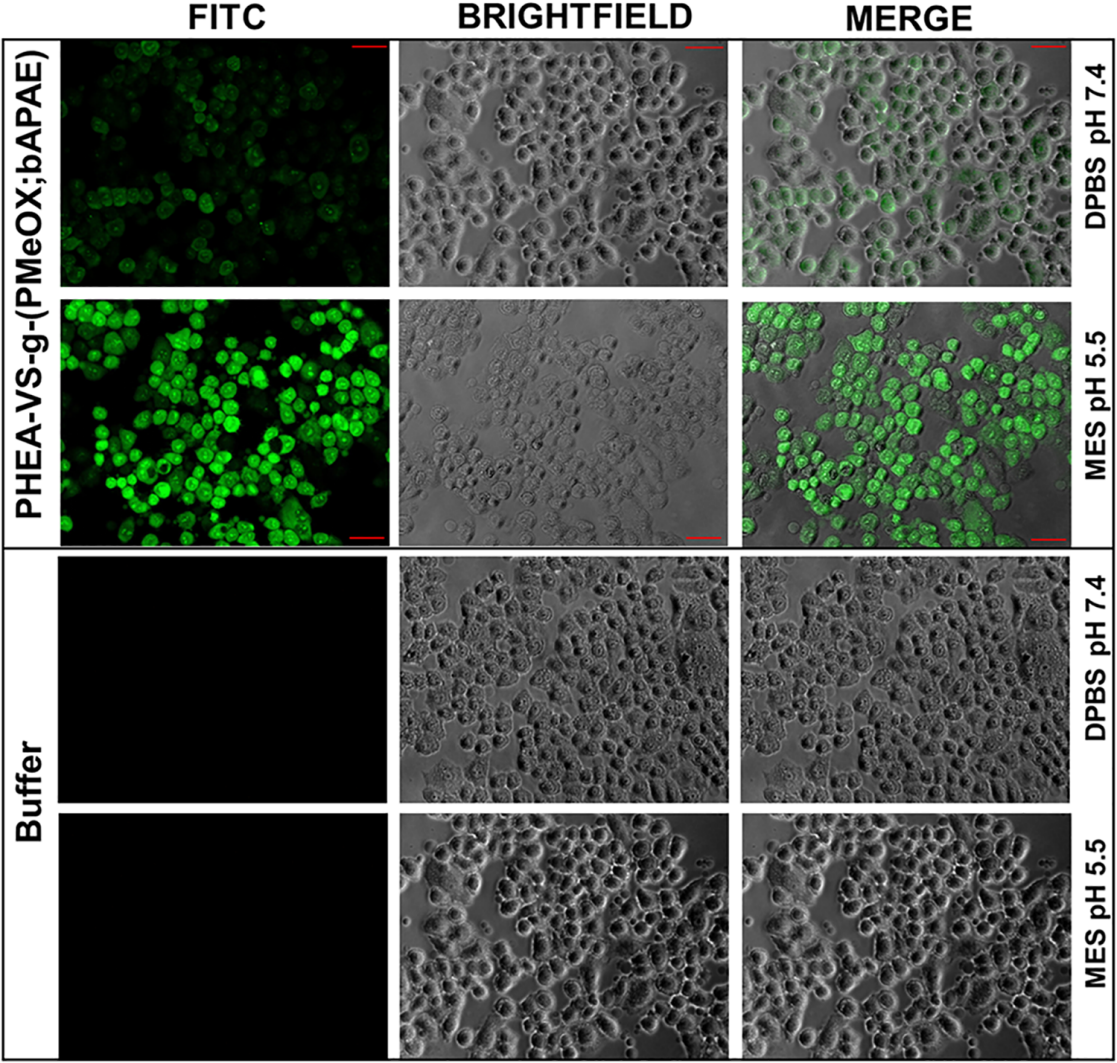
Fluorescence images of 16-HBE cells after incubation at pH 7.4 and 5 with (upper) and without (lower) PHEA-VS-*g*-(PMeOx;bAPAE) graft copolymer.

Overall, these results suggest that the copolymer preferentially interacts with cellular membranes under acidic conditions, consistent with a mechanism that mimics endosomal/lysosomal membrane destabilization and is likely driven by the pH-dependent protonation of the polymer. Therefore, the strategy of exploiting VS residues for conjugating the tetramine to the polymer backbone enhanced the buffering properties, as it allowed the retention of all amine groups for each functionalized RU.^9^

### 2.2 Polyplex Production and Characterization

The ability of PHEA-VS-*g*-(PMeOx;bAPAE) to complex siRNA was tested by mixing equal volumes of siRNA (0.2 mg/mL) and copolymer at varying concentrations to achieve polymer/siRNA weight ratios (R) from 0 to 7. Polyplex formation was assessed by agarose gel electrophoresis (Figure 4a) and DLS (Figure 4b). Stable complexes formed from R5 (N/P ∼ 1.65 at pH 7.4), with zeta potentials shifting from slightly negative at R1 to slightly positive at R10, and near-neutral at R5, reflecting the copolymer design that favors small, nearly neutral nanoparticles at low pH. Particle sizes remained below 40 nm from R5 to R10, indicating minimal aggregation, likely due to steric stabilization by surface PMeOx chains. AFM imaging at R10 (Figure 4c) confirmed the formation of nanometric polyplexes. Furthermore, by evaluating the siRNA release capacity, it was demonstrated that the complexes are stable and that this stability increases with increasing R (Figure S6).

**Figure 4:**
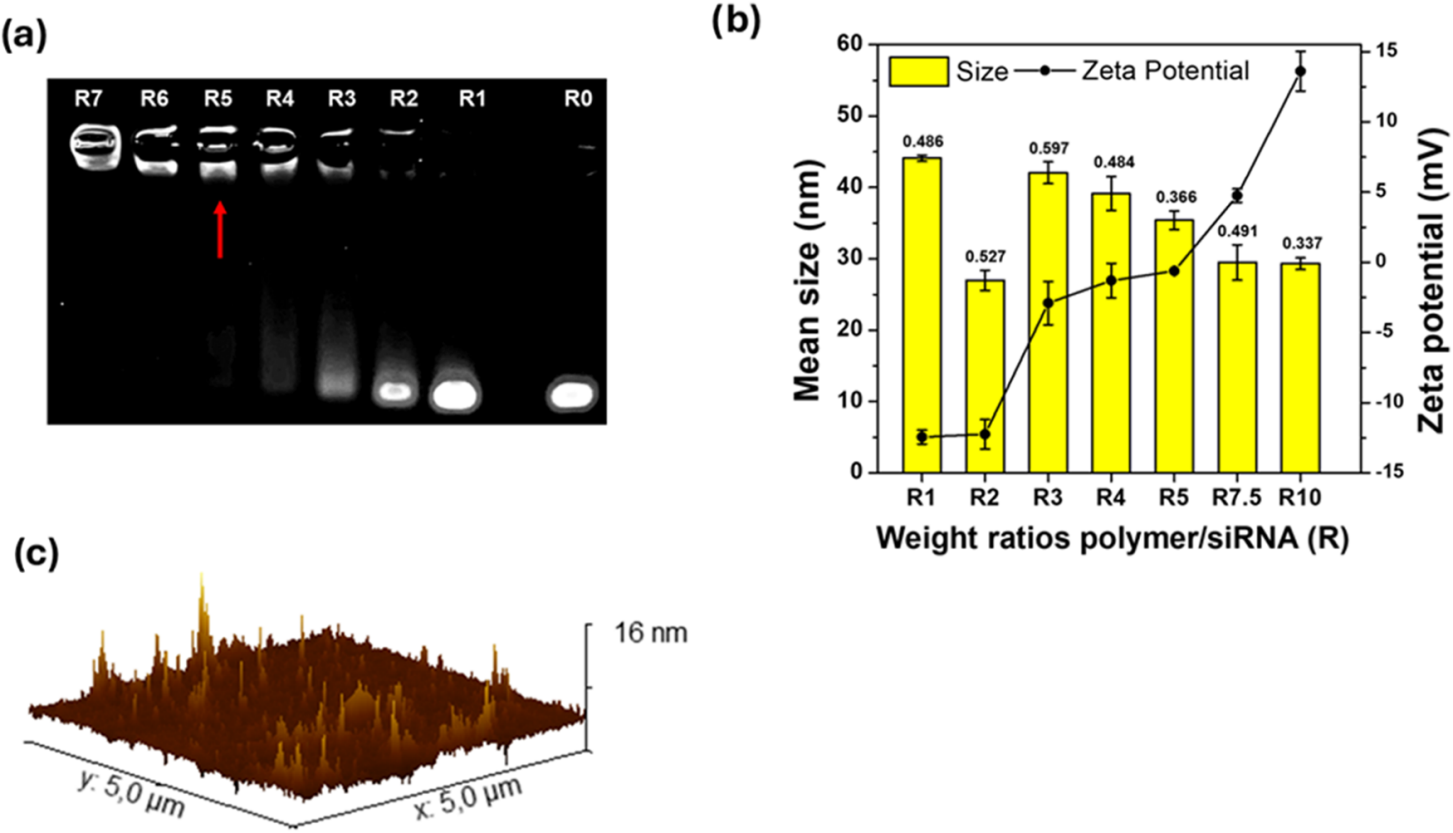
(a) Agarose gel electrophoresis of PHEA-VS-*g*-(PMeOx;bAPAE)/siRNA polyplexes obtained in PBS at various copolymer to siRNA weight ratios (R); (b) mean size (istogram), PDI values (data labels) and zeta potential (continued line) of PHEA-VS-*g*-(PMeOx;bAPAE)/siRNA polyplexes measured by DLS in Hepes buffer (pH 7.4) at various weight ratios (R) (data are reported as means ± SD, *n* = 3); (c) AFM image of polyplexes at R10.

Overall, these results confirm the potential of the proposed material as an siRNA delivery vector. In a previous study,^9^ we developed a platform also based on PHEA and bAPAE; however, it was obtained through a different synthetic protocol and employed PEG to enhance hydrophilicity. Comparison between the two polymers shows that the material here described exhibits a higher complexation ability (N/P ∼1.65 vs ∼3), despite a lower amine content (DD% bAPAE ∼25% vs ∼35%). This results in the formation of polyplexes with significantly smaller sizes, approximately one-third of those previously obtained (∼40 nm vs ∼110 nm).

### 2.3 Stability Studies

The stability of PHEA-VS-*g*-(PMeOx;bAPAE) polyplexes for pulmonary delivery was tested in the presence of lung surfactant. Agarose gel electrophoresis showed that polyplexes at R5 and R10 remained intact after 5 h in Curosurf, with no siRNA release (Figure 5a). Protection against RNase A was confirmed, with ∼70% siRNA recovered at R5 and ∼100% at R10, while free siRNA was completely degraded (Figure 5b).

**Figure 5:**
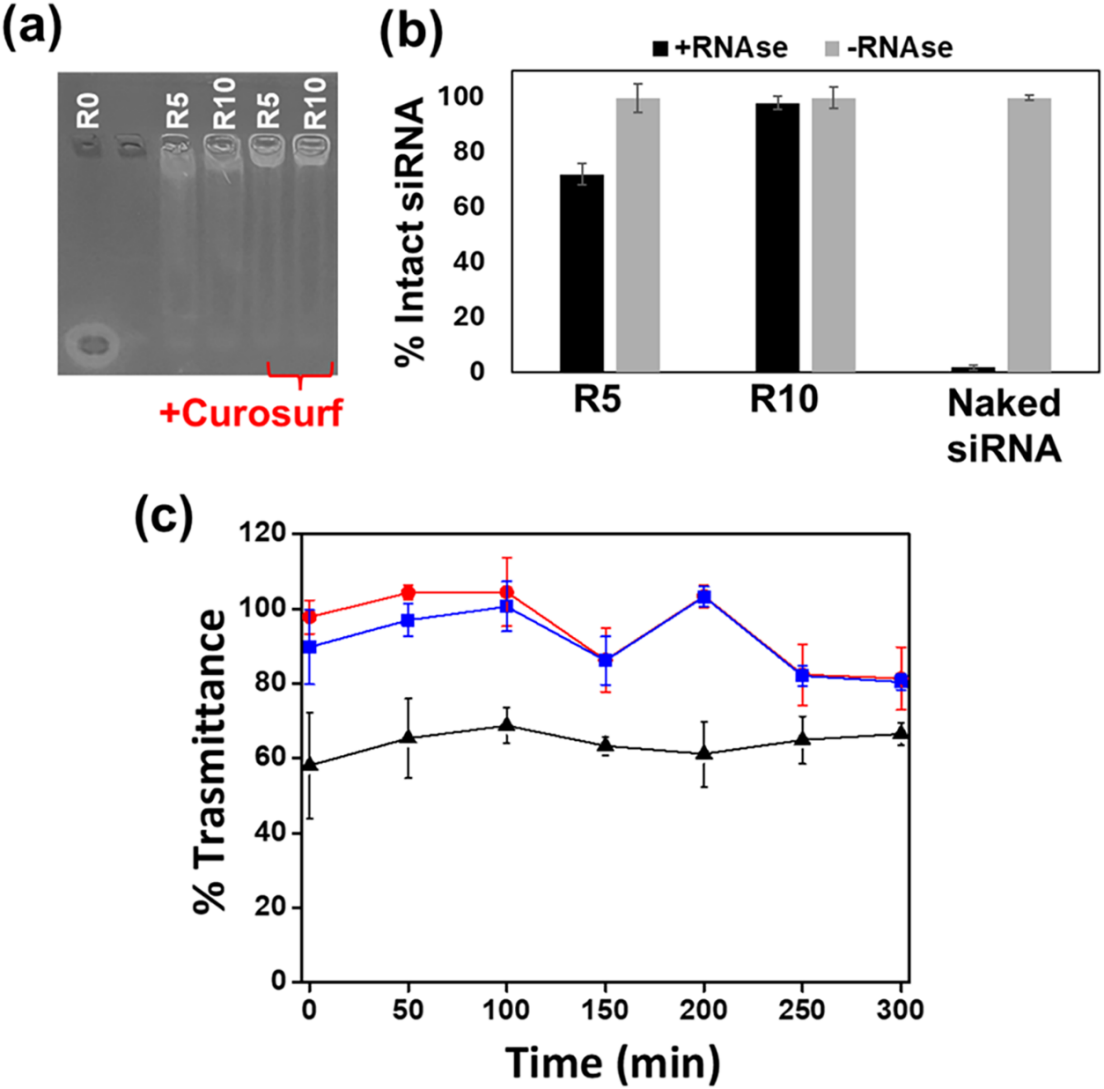
Evaluation of the electrophoretic mobility of siRNA in the polyplexes after 5 h of incubation with and without Curosurf (a); quantification of siRNA after 1 h of incubation with RNase (b); transmittance at 500 nm of dispersions containing mucin in the presence of polyplexes R10 (red), polyplexes R10 + Pen (blue) and chitosan (black) (c).

Since mucins, the main glycoproteins in lung mucus, carry negatively charged sulfate and sialic acid residues,^46^ the potential for aggregation of positively charged polyplexes was assessed by turbidimetry at R10, using chitosan as a positive control. Transmittance remained high over time, indicating that polyplexes were stable and did not aggregate with mucin, whereas chitosan caused a marked decrease in transmittance over 6 h (Figure 5c). These results demonstrate that PHEA-VS-*g*-(PMeOx;bAPAE) polyplexes efficiently complex siRNA at low R values, remain stable in lung-relevant environments, and protect siRNA from nuclease degradation, supporting their potential for inhalation-based delivery.

### 2.4 Preparation and Characterization of Penetratin (Pen) Decorated Polyplexes

To enhance the intracellular delivery of siRNA, polyplexes were surface-decorated with Penetratin (Pen), a cell-penetrating peptide (CPP) widely used in gene and drug delivery due to their ability to promote internalization of cargos, and facilitate endosomal escape, with low cytotoxicity.^18,19^ Formed polyplexes were surface-functionalized with a Pen, bearing N-terminal azido-lysine residue, which enabled site-specific conjugation to the alkyne-terminated PMeOx side chains of the PHEA-VS-*g*-(*pro*PMeOx;bAPAE) copolymer. This strategy allowed for efficient and stable conjugation of the peptide onto the polyplex surface, while preserving the structural integrity of both siRNA and peptide. This reaction was carried out via copper­(I)-catalyzed azide–alkyne cycloaddition (CuAAC), also known as the Huisgen “click” reaction, using copper­(II) sulfate (CuSO_4_) and ascorbic acid as catalysts. A schematic representation of this concept is shown in Figure 6.

**Figure 6:**
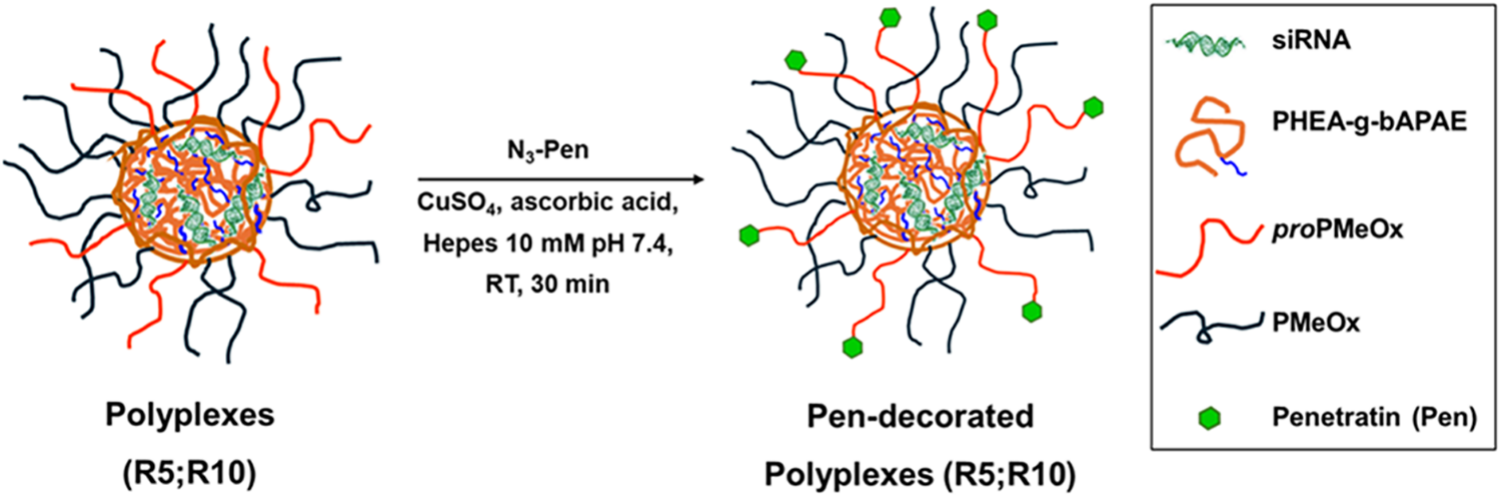
Schematic representation of surface decoration of formed polyplexes with Pen via click chemistry.

To obtain polyplexes with different Pen surface density, these were produced at R5 and R10 by using a polymeric dispersion of a mixture 99:1 w/w of PHEA-VS-*g*-(PMeOx;bAPAE) and PHEA-VS-*g*-(*pro*PMeOx;bAPAE). HPLC analysis of the unbound peptide demonstrated that the functionalization reaction proceeded efficiently for about 100%, for both R5 and R10 polyplexes, suggesting a complete saturation of the alkyne-terminated *pro*PMeOx by the azide Pen derivative.

DLS analysis showed that surface decoration of polyplexes with Pen did not affect their colloidal stability, with R5 and R10 polyplexes maintaining ∼40 nm particle size and zeta potentials similar to undecorated systems (data not shown). Turbidimetric measurements at R10 in mucin dispersions (Figure 5c) confirmed that Pen decoration did not induce aggregation, indicating that the physicochemical properties of the polyplexes remained unchanged.

### 2.5 Biological Characterization

Polyplex compatibility with airway epithelial cells was evaluated in vitro using human bronchial epithelial (16-HBE) cells, a relevant model for inhalation exposure and airway inflammation.^47^ Cell viability, assessed by MTS assay after 24 and 48 h exposure, remained above 80% for the PHEA-VS-*g*-(PMeOx;bAPAE) copolymer at concentrations up to 0.5 mg/mL (Figure 7a). Polyplexes at R5 and R10, with or without Pen decoration (200 nM siRNA), also showed high viability after both time points (Figure 7b), confirming their cytocompatibility and potential safety for therapeutic use.

**Figure 7:**
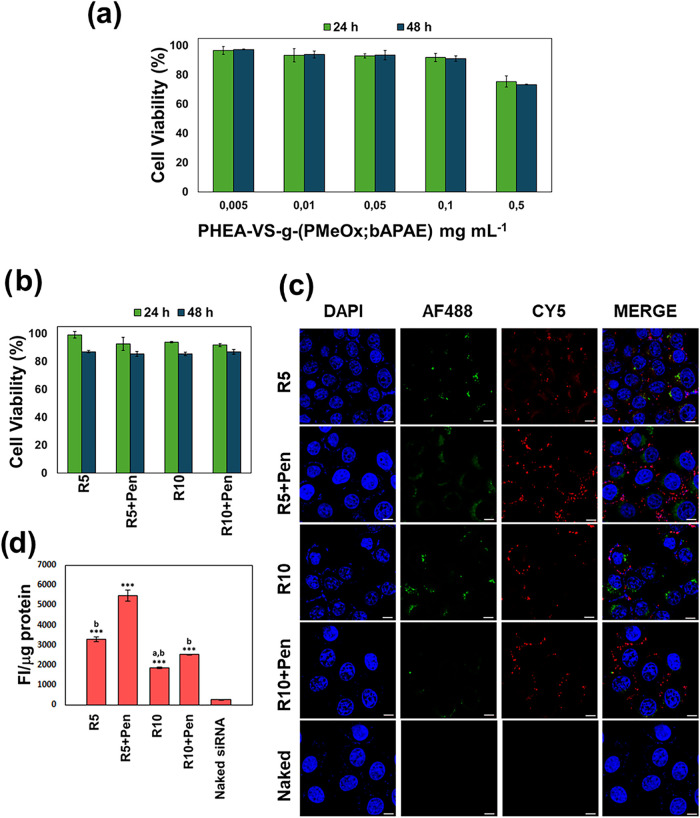
Cell viability of 16-HBE cells treated with (a) PHEA-VS-*g*-(PMeOx;bAPAE) graft copolymer at concentrations ranging between 0.005 and 0.5 mg mL^–1^; (b) polyplexes at R5 and R10, with and without surface decoration with Pen, after 24 and 48 h incubation (final siRNA concentration = 200 nM); (c) CLSM images of 16-HBE cells treated for 24 h with PHEA-VS-*g*-(PMeOx;bAPAE)_Alexafluor488_/siRNA_Cy5_ polyplexes at R5 and R10, with and without surface decoration with Pen, and naked siRNA_Cy5_ (bar represents 10 μm); (d) quantitative cellular uptake, expressed as FI/μg of protein, of 16HBE cells treated after 24 h of incubation with PHEA-VS-*g*-(PMeOx;bAPAE)_Alexafluor488_/siRNA_Cy5_ polyplexes at R5 and R10, with and without Pen peptide, and naked siRNA. Data are expressed as means ± SD (*n* = 3) where ***p < 0.001 vs siRNA naked, ^a^ *p* < 0.05 vs polyplexes R5, ^b^ *p* < 0.05 vs polyplexes R5 + Pen.

To study the process uptake of polyplexes into cells, an in vitro study was assessed on 16-HBE cells after 24 h incubation by using fluorescent PHEA-VS-*g*-(PMeOx;bAPAE)_Alexafluor488_/siRNA_Cy5_ polyplexes, produced by labeling PHEA-VS-*g*-(PMeOx;bAPAE) and siRNA, respectively, with AlexaFluor488 (green) and Cy5 (red). Confocal laser scanning microscopy (CLSM) (Figure 7c) revealed intracellular fluorescence signals for both the labeled copolymer and siRNA, with partial colocalization of these signals, indicating siRNA release. In contrast, no fluorescence signal was observed in cells treated with naked siRNA, highlighting its poor cellular internalization and therefore significant improvement of siRNA cell uptake was found thanks to the complexation with the copolymer.

The quantification of intracellular fluorescence due to siRNA_Cy5_ internalization, expressed as FI showed in Figure 7d, confirmed that siRNA_Cy5_ uptake is significantly higher when it is complexed with the copolymer compared to free siRNA _Cy5_. Moreover, Pen surface decorated polyplexes at both R5 and R10 are significantly cell uptaken, with polyplexes formulated at R5 showing the highest uptake levels (∼5600 FI/μg). This result could be explained by considering that the polyplexes at R10, display a greater amount of PMeOx on their surface than that obtained at R5, which could reduce the interactions between the polyplexes and the cell membranes, due to the formation of a more compact hydrophilic shell. After confirming their cellular internalization, the endosomal escape of the obtained polyplexes was subsequently evaluated. This process is essential to ensure the cytoplasmic bioavailability of the delivered siRNA, a critical prerequisite for effective gene-silencing activity. Following incubation with the polyplexes, cells were analyzed by confocal laser scanning microscopy (CLSM) to qualitatively assess the cytosolic release of siRNA (Figure S7). The Pearson’s correlation coefficient (PCC), which measures the degree of colocalization between the two fluorescence channels, ranged from 0.46 to 0.70 across all samples. These values indicate partial colocalization, suggesting successful escape from endosomal compartments. This behavior supports enhanced cytoplasmic availability of siRNA and highlights the potential of these polyplexes for efficient gene silencing.

### 2.6 Gene Silencing

Once confirmed the Pen surface good cytocompatibility and efficient internalization by 16-HBE of decorated polyplexes, their gene silencing ability was assessed in vitro by using MDA-MB-231 cells, stably expressing the luciferase reporter gene. These cells were treated with siRNA, free naked or scrambled (siGL2/3 or siNC, respectively) or with Pen decorated polyplexes (R5 and R10). siRNA/Turbofect was used as positive control. The extent of gene silencing was determined by calculating the relative luciferase activity (expressed as % RLU per mg of protein in treated cells relative to that in untreated control cells), and the results are shown in Figure 8a. The obtained results show that surface modification with Pen achieves approximately 50% inhibition for polyplexes prepared at R5 and around 30% inhibition at R10, associated with high cell viability (Figure 8). This result is in accordance with cell uptake studies (see Figure 7b and 7c). On the contrary, unmodified polyplexes exhibit poor silencing efficiency (Figure S8).

**Figure 8:**
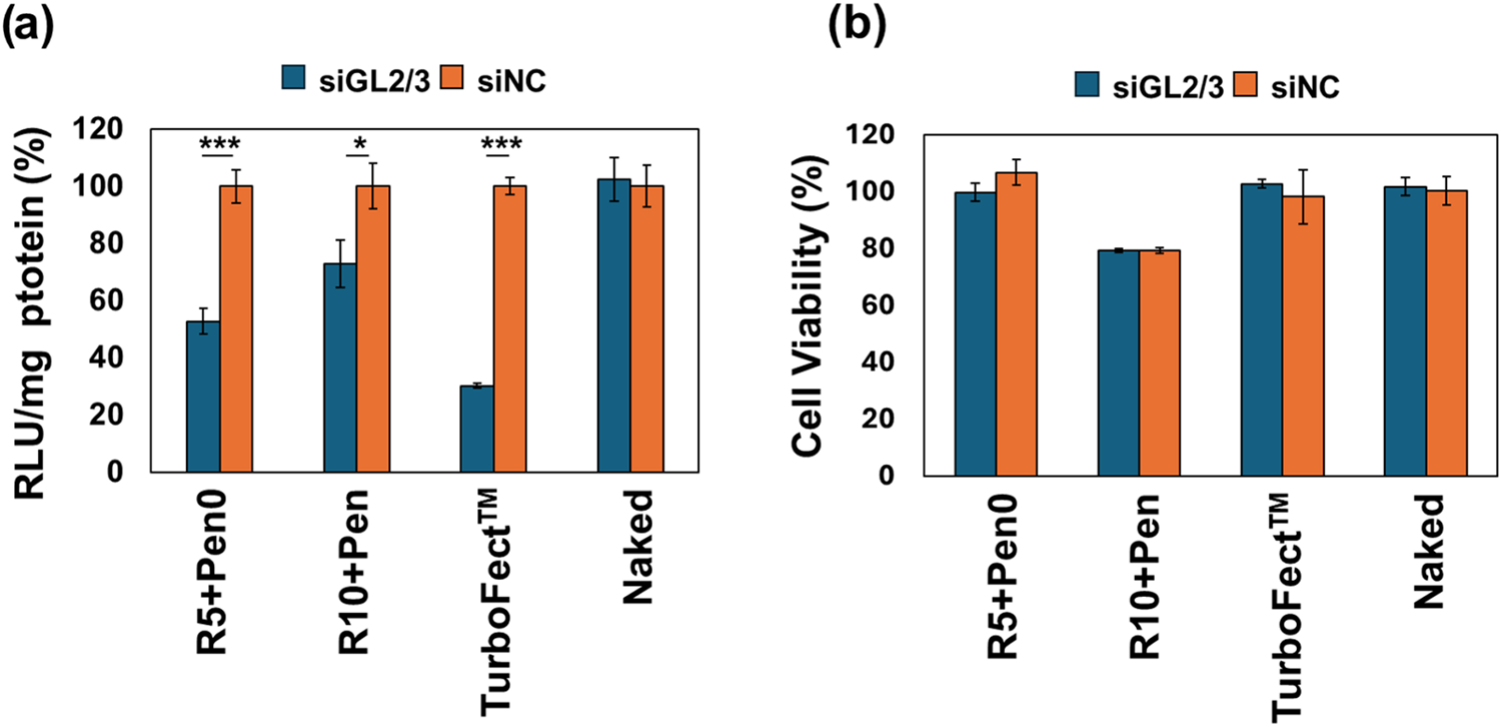
(a) Gene silencing efficiency and (b) cell viability of MDA-MB-231 cells after 72 h of incubation with various formulations containing either free or complexed siGL2/3 (red) or scrambled siRNA (siNC, blue) at a final concentration of 200 nM siRNA per well. Data are expressed as means ± SD (*n* = 3). *p < 0.05, ***p < 0.001.

Overall, these results demonstrate that the PHEA-VS-*g*-(PMeOx;bAPAE)-based carrier, functionalized with the cell-penetrating peptide Pen, achieves a remarkably high effect of gene silencing in vitro. This outcome reflects not only efficient siRNA internalization, but also effective endosomal escape and cytosolic release, which remain major limitations of nonviral vectors. Therefore, the observed silencing efficiency highlights the capability of this polymeric platform to overcome key intracellular barriers, underscoring its strong potential as a safe and effective polymeric carrier for siRNA delivery, particularly in lung-targeted applications.

### 2.7 In Vitro Pulmonary Drug Deposition

To assess the feasibility of pulmonary administration of the polyplexes via nebulization, an aerosolization study was conducted using an Andersen Cascade Impactor (ACI) on an aqueous dispersion of polyplexes at R10. As shown in Figure 9, the deposition profile demonstrated that approximately 75% of the nebulized polyplexes were efficiently deposited between stages 3 and 7, corresponding to the bronchiolar and alveolar regions, which are critical targets for pulmonary gene therapy.

**Figure 9:**
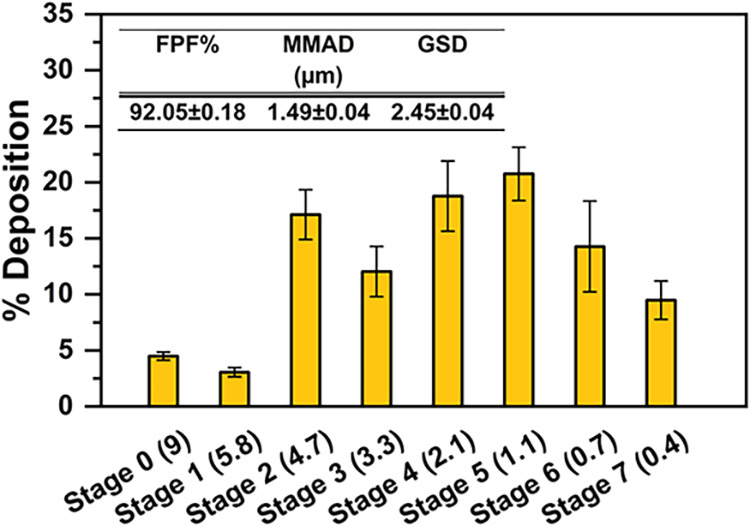
Deposition of polyplexes aqueous dispersion at R10 on the stages of the ACI.

Moreover, the formulation exhibited excellent aerosolization performance, with an exceptionally high fine particle fraction (FPF) of 92.05 ± 0.18%, indicating a strong propensity for deep lung deposition. In addition, the mass median aerodynamic diameter (MMAD) of 1.49 ± 0.04 μm places the formulation well within the optimal respirable range. Altogether, these aerodynamic parameters highlight the robustness of the formulation during nebulization and strongly support the suitability of the PHEA-based polyplexes as a promising noninvasive platform for effective siRNA delivery to the lower respiratory tract.

## 3 Conclusions

In this work, we successfully developed and thoroughly characterized a synthetic copolymer based on α,β-poly­(*N*-2-hydroxyethyl)-d,l-aspartamide/poly­(2-methyl-2-oxazoline)/1,2-Bis­(3-aminopropylamino)­ethane, namely PHEA-VS-*g*-(PMeOx;bAPAE), specifically engineered for the pulmonary delivery of siRNA via inhalation. The employed synthetic route is efficient and adaptable, utilizing prior functionalization with divinylsulfone (DVS) to enable a one-pot reaction that simultaneously introduces hydrophilic PMeOx chains and cationic amino groups. This method results in a high-yield copolymer featuring a well-balanced architecture combining polycationic and hydrophilic domains. The copolymer displays favorable physicochemical properties, with amine-rich regions essential for siRNA binding, and PMeOx segments that enhance hydrophilicity and prevent aggregation under physiological conditions. Our findings confirm that the copolymer forms stable complexes with siRNA at polymer-to-siRNA weight ratios (R) of 5, generating polyplexes with diameters below 40 nm. Such nanoscale dimensions are critical for effective transport across the mucus layer and periciliary fluid in the lungs. Moreover, the copolymer’s buffering capacity supports efficient endosomal escape via the proton sponge mechanism, a key step for successful intracellular delivery of siRNA. The observed pH-dependent membrane destabilization further suggests enhanced cytosolic release, thus increasing the bioavailability of the therapeutic agent. The polyplexes demonstrate high resistance to siRNA premature release in the presence of pulmonary mucus and surfactant, confirming their suitability for aerosol-based administration. Additionally, the system provides substantial protection to siRNA against enzymatic breakdown, extending its bioactivity after delivery. Surface functionalization of polyplexes with penetratin (Pen), a cell-penetrating peptide (CPP), by exploiting the terminal alkyne groups on the PMeOx chains, promotes increased siRNA internalization by bronchial epithelial cells (16-HBE), compared to free siRNA. These results are corroborated by in vitro gene silencing experiments targeting luciferase in MDA-MB-231 cells, showing effective downregulation of gene expression, with highest activity showed by the Pen surface decorated polyplexes obtained with R5. Considering also the excellent aerosolization properties of aqueous dispersion of polyplexes, the PHEA-VS-*g*-(PMeOx;bAPAE) graft copolymer represents a highly promising siRNA delivery platform for pulmonary applications due to the minimal interaction with mucus components and ability to form ultrasmall, stable polyplexes for inhalation-based treatment strategies aimed at lung diseases.

## 4 Experimental Section

### 4.1 Materials

Dimethylformamide (DMF), methanol (MeOH), diethyl ether, dichloromethane, acetone, ascorbic acid, copper­(II) sulfate (CuSO_4_), triethylamine (TEA), divinyl sulfone (DVS), benzonitrile, Dulbecco’s phosphate-buffered saline (DPBS), Hepes buffer, sodium hydroxide (NaOH), hydrochloric acid (HCl), sodium chloride (NaCl), agarose, 2-methyl-2-oxazoline (MeOX), propargyl p-toluenesulfonate (*pro*Tos), BOC-piperazine, 1,2-bis­(3-aminopropylamino)­ethane (bAPAE), mucin from porcine stomach (Type II), RNase A, and the MISSION siRNA Fluorescent Universal Negative Control #1 labeled with Cyanine 5 were obtained from Merck (Italy). Methyl trifluoromethanesulfonate (MeOTf) were obtained from Perlabo (Italy). Silencer Negative Control No. 1 siRNA and Silencer GL2/3 siRNA, AlexaFluor 488 NHS ester were purchased from Thermofisher (Italy). Penetratin (Pen) [Lys (N_3_)­RQIKIKFQNRRMKWKK] was purchased from Bio-Fab (Italy). The hydroquinone present in the commercially available divinyl sulfone ≥ 98.0% (used as stabilizing agent) was removed through neutral aluminum oxide column before use. Benzonitril, MeOx, PTos and MeOTf were purified by treatment with calcium hydride, followed by vacuum distillation, and stored under appropriate inert conditions. All materials used for biological characterization were purchased from Merck (Italy). α, β-poly­(*N*-2-hydroxyethyl)-d,l-aspartamide (PHEA) was synthesized and purified as previously reported.^33^

### 4.2 Cell Culture

Human bronchial epithelial (16-HBE) cells were obtained from the Istituto Zooprofilattico Sperimentale della Lombardia e dell’Emilia-Romagna and cultured in DMEM supplemented with 10% FBS, 2 mM l-glutamine, amphotericin B (2.5 μg/mL), streptomycin (100 μg/mL), and penicillin (100 U/mL) (Sigma-Aldrich). Luciferase-expressing MDA-MB-231 breast cancer cells (CliniSciences S.r.l.) were maintained under the same conditions, with 0.6 μg/mL puromycin. All cells were incubated at 37 °C with 5% CO_2_ and 95% humidity.

### 4.3 Methyl-poly­(2-methyl-2-oxazoline) (PMeOx) and propargyl-poly­(2-methyl-2-oxazoline) (proPMeOx) Synthesis by Cationic Ring-Opening Polymerization (CROP)

Poly­(2-methyl-2-oxazoline) with an average molecular weight of approximately 5 kg/mol was prepared following procedures described in previous literature.^30,48,49^ In brief, 10.7 g of 2-methyl-2-oxazoline (MeOx, 58 equiv) were added to a dried flask under argon with 42 mL benzonitrile (∼3 M). Then, 360 mg methyl triflate (MeOTf, 1 equiv) or 425.91 mg p-toluenesulfonate (proTos) was added, and the mixture was stirred at 120 °C for 3 h. After cooling, 1.23 g 1-Boc-piperazine (3 equiv) in 3.5 mL benzonitrile was added for end-capping at 50 °C overnight. The polymer was precipitated in cold diethyl ether (0 °C), centrifuged, washed twice with fresh ether, and dried under reduced pressure. The polymer was obtained in ∼97% yield and characterized by ^1^H NMR and SEC.

^1^H NMR PMeOx-Pip-Boc (300 MHz, **CDCl**_**3**_, 25 °C, TMS) δ: 1.44–1.46 (m, 9H, (C**H**_**3**_)_3_CO), 2.07–2.13 (m, 174H, [C**H**_**3**_CON]-), 2.94, 3.03–3.05 (m, 3H, C**H**_**3**_ **[**N–CH_2_CH_2_]-), 3.45–3.47 (m, 232H [-C**H**_**2**_C**H**_**2**_N-]).

^1^H NMR *pro*PMeOx-Pip-Boc (300 MHz, **CDCl**_**3**_, 25 °C, TMS) δ: 1.43 (m, 9H, (C**H**_**3**_)_3_CO), 2.07–2.13 (m, 174H, [C**H**_**3**_CON]-), 3.43–3.47 (m, 232H [-C**H**_**2**_C**H**_**2**_N-]).

The BOC protecting group was removed in acidic medium as already reported,^30,48^ and the recover product product, obtained with an approximate yield of 98% (w/w based on the starting polymer), was characterized by ^1^H NMR analysis and SEC analyses.

### 4.4 Synthesis of PHEA-Vinylsulfone (PHEA-VS)

A total of 1 g of PHEA, corresponding to 6.33 mmol of repeating units, was dissolved in 20 mL of dimethylformamide (DMF), then subsequently 3.2 mL of purified DVS (31.6 mmol) and 4.4 mL of TEA (31.6 mmol) were added dropwise. The reaction mixture was stirred at 60 °C for 48 h under protection from light. After completion of the reaction, the solution was slowly poured into 200 mL of a diethyl ether/acetone mixture (1:1, v/v) to induce precipitation. The resulting suspension was centrifuged, and the precipitate was washed five times with the same ether/acetone mixture. The purified solid was then redissolved in 15 mL of ultrapure water, filtered using a 0.22 μm cellulose acetate syringe filter (Sartorius, Minisart, Germany), and subsequently freeze-dried under protection from light. The final product (PHEA-VS) was obtained with a yield of 92% by weight relative to the initial amount of PHEA. Characterization was carried out by ^1^H NMR analysis and SEC analyses.

^1^H NMR PHEA-VS (400 MHz, **D**_**2**_ **O**, 25 °C, TMS) δ: 2.84 (m, 2H_PHEA_, −COCHC**H**_**2**_CONH−), 3.24 (m, 2H_PHEA_, -NHC**H**_**2**_CH_2_O−), 3.55 (m, 2H_PHEA_, -NHCH_2_C**H**_**2**_OH), 4.59 [m, 1H_PHEA_, -NHC**H**(CO)­CH_2_−], 6.37–6.50 (m, 2H_VS_, C**H**_**2**_=) and 6.9 (m, 1H_VS_, = C**H**-).

### 4.5 Synthesis of PHEA-VS-*g*-(PMeOx;bAPAE) and PHEA-VS-*g*-(proPMeOx;bAPAE)

An amount of 300 mg of PHEA-VS (corresponding to 1.434 mmol of repeating units) was dissolved in 3 mL of ultrapure water. Once fully solubilized, 395 mg of PMeOx or *pro*PMeOx, previously dissolved in 1.5 mL of ultrapure water, were added. The pH of the solution was adjusted to 10 using 1 M NaOH, and the reaction mixture was stirred overnight in the dark. The following day, the reaction mixture was mixed with a bAPAE solution (1.1 g dissolved in 75 mL of DMF) and allowed to stir overnight under light-protected conditions. Then, the reaction volume was reduced to approximately one-third using a rotary evaporator and then precipitated dropwise into 250 mL of a diethyl ether/dichloromethane (2:1, v/v) mixture. The resulting precipitate was collected by centrifugation and washed five times with acetone. The purified solid was dried under vacuum, redissolved in 5 mL of ultrapure water, dialyzed (MWCO 3.5 kDa), and finally freeze-dried. For fluorescent labeling, 1 mL of a 10 mg/mL solution of PHEA-VS-*g*-(PMeOx;bAPAE) in phosphate-buffered saline (PBS, pH 8.3) was mixed with 28 μL of Alexa Fluor 488 5-SDP Ester (2 mg/mL in DMSO). The mixture was incubated at room temperature for 1 h. After the labeling reaction, the polymer was purified via dialysis (MWCO 3.5 kDa) and subsequently freeze-dried.

^1^H NMR *PHEA-VS-g-(PMeOx;bAPAE)* (400 MHz, **D**_**2**_ **O**, pD < 5, 25 °C, TMS): δ 2.11 (m, 4H_bAPAE_, -NHCH_2_C**H**_**2**_CH_2_NHCH_2_CH_2_NHCH_2_C**H**_**2**_CH_2_NH; 174H_PMeOx_, -[C**H**_**3**_CON]-), 2.84 (m, 2H_PHEA_, −COCHC**H**_**2**_CONH−), 3.05–3.15 (m, 12H_bAPAE_, -NHC**H**_**2**_CH_2_C**H**_**2**_NHC**H**_**2**_C**H**_**2**_NHC**H**_**2**_CH_2_C**H**_**2**_NH_2_), 3.56 (m, 232H_PMeOx_ [-C**H**_**2**_C**H**_**2**_N-]), 3.67 (m, 2H_PHEA_, -NHCH_2_C**H**_**2**_OH), 4.0 (m, 2H_PHEA_, -NHCH_2_C**H**_**2**_OCH_2_CH_2_–S-), 4.7 (m, 1H_PHEA_, -NHC**H**(CO)­CH_2_−).

^1^H NMR *PHEA-VS-g-(PMeOx;bAPAE)* (400 MHz, **D**_**2**_ **O**, pD > 10, 25 °C, TMS) δ: 1.7 (m, 4 H_bAPAE_, NHCH_2_C**H**_**2**_CH_2_NHCH_2_CH_2_NHCH_2_C**H**_**2**_CH_2_NH), 2.11 (m, 174H_PMeOx_,-[C**H**_**3**_CON]-), 2.69 (m, 12H_bAPAE_, NHC**H**_**2**_CH_2_C**H**_**2**_NHC**H**_**2**_C**H**_**2**_NHC**H**_**2**_CH_2_C**H**_**2**_NH_2_; m, 2H_PHEA_, −COCHCH_2_CONH−), 3.56 (m, 232H_PMeOx_ [-C**H**_**2**_C**H**_**2**_N-]), 3.67 (m, 2H_PHEA_, -NHCH_2_C**H**_**2**_OH), 4.0 (m, 2H_PHEA_, -NHCH_2_C**H**_**2**_OCH_2_CH_2_–S-), 4.7 (1 H_PHEA_, m: NHC**H**(CO)­CH_2_−).

### 4.6 Size Exclusion Chromatography (SEC)

Size exclusion chromatography (SEC) was performed on a PolySep-GFC-P4000 column (Phenomenex) at 30 °C, using an Agilent 1260 Infinity Multi-Detector GPC/SEC system with a refractive index detector. The mobile phase was 0.15 M citrate/phosphate buffer (pH 5) at 0.8 mL/min. Molecular weight distributions were determined via calibration with poly­(ethylene oxide) standards.

### 4.7 Potentiometric Titration of INU-VS-*g*-(PMeOx;bAPAE)

A 30 mL solution of PHEA-VS-*g*-(PMeOx;bAPAE) at a concentration of 1 mg mL^–1^ was titrated under an argon atmosphere using 0.05 N HCl until the pH reached 3. Subsequently, the same sample was titrated back with 0.05 N NaOH. An equivalent procedure was applied to a comparable amount of bAPAE for reference. To maintain constant ionic strength, the samples were dissolved in 0.1 N degassed aqueous sodium chloride solution. Prior to the titrations, the Jenway differential electrometer was calibrated using a series of standard buffer solutions covering a pH range from 2.50 ± 0.01 to 10.00 ± 0.01.

### 4.8 Membrane Destabilization Study

16-HBE cells were plated at 10,000 cells/well in 8-well Nunc Lab-Tek chambers. The next day, the medium was replaced with 100 μL of PHEA-VS-*g*-(PMeOx;bAPAE) (5 μg/mL) in either DPBS (pH 7.4) or 20 mM MES buffer (pH 5.5, 130 mM NaCl). After 20 min incubation at 37 °C, cells were washed with DPBS, stained with 1 μM Oxazole yellow for 10 min at 37 °C, and fixed with 4% formaldehyde. Imaging was performed using an inverted epifluorescence microscope (Axio Cam MRm, Zeiss), and images were analyzed with AxioVision software. Controls used only the respective buffers under the same conditions.

### 4.9 Polyplex Assembly

Polyplexes were obtained starting from equal volumes of siRNA (0.2 mg mL^–1^ in RNase-free water) and copolymer at various concentrations in 20 mM HEPES buffer (pH 7.4), to obtain a range of polymer/siRNA weight ratios (R) going from 1 to 7, which were properly mixed. The extent of complex formation was assessed using gel retardation assays and Dynamic Light Scattering (DLS) analysis, as previously described.^9^ For gel retardation assays, glucose was added to 20 mM HEPES buffer pH 7.4 to obtain a 10% (w/v) solution. DLS analysis was also performed using copolymer at various concentrations in 20 mM Acetate buffer (pH 5.5).

### 4.10 Atomic Force Microscopy (AFM)

AFM micrographs were obtained on a FAST-SCAN microscope equipped with a closed-loop scanner (*X*, *Y*, and *Z* maximum scan region: 35, 35, and 3 μm, respectively). The analysis was performed in soft tapping mode using a probe with an apical radius of 5 nm operating at 1400 kHz (k: 18 N/m).

### 4.11 Scanning Transmission Electron Microscope (STEM)

the morphology of polyplexes was determined by scanning transmission electron microscopy (STEM). To prepare the STEM grids, one drop of sample dispersion (1 mg/mL) was placed on a holey carbon-coated copper grid, air-dried overnight, and imaged using an SEM/STEM Fei-ThermoFisher Versa 3D.

### 4.12 Effect of Pulmonary Surfactant

The stability of polyplexes in the presence of pulmonary surfactant was evaluated by agarose gel electrophoresis, following previously reported protocol.^50^ Polyplexes (20 μL) at polymer/siRNA weight ratios (R) of 5 and 10 were incubated with 5 μL of Curosurf at 37 °C for 5 h. Electrophoresis was then performed as in the complexation assay. As a control, surfactant was replaced with 10 mM nuclease-free HEPES buffer (pH 7.4).

### 4.13 Effect of RNase

Protection of siRNA against enzymatic digestion was evaluated by incubating the polyplexes with RNase A. Briefly, 10 μL of polyplexes (polymer/siRNA weight ratios 5 or 10, containing 2 μg siRNA) were treated with 2 μL of RNase A solution (20 μg mL^–1^) and incubated at 37 °C for 1 h. Samples were then heated at 95 °C for 10 min, cooled, and supplemented with 4 μL of heparin (1000 IU mL^–1^), followed by an additional 10 min incubation. The remaining intact siRNA was quantified using the RediPlate 96 RiboGreen RNA Kit. Control samples were processed in parallel by replacing RNase A with nuclease-free 10 mM HEPES buffer (pH 7.4).

### 4.14 In Vitro Release

To evaluate the in vitro release profiles, 400 μL of polyplexes prepared at charge ratios (R) of 5 and 10 were loaded into dialysis tubes (MWCO 50 kDa) and immersed in 5.6 mL of 10 mM HEPES buffer (pH 7.4) to maintain sink conditions. At predetermined time intervals, 100 μL of the external (acceptor) medium was withdrawn and replaced with an equal volume of fresh buffer. Each collected aliquot was analyzed using the SYBR Gold assay to quantify the released siRNA. Samples were incubated in the dark with 30 μL of 4× SYBR Gold Nucleic Acid Gel Stain, and fluorescence was measured using a microplate reader at an excitation wavelength of 492/20 nm and an emission wavelength of 537/20 nm. As a control, the diffusion profile of an equivalent amount of free siRNA was evaluated under the same conditions.

### 4.15 Preparation and Characterization of Penetratin (Pen)—Decorated Polyplexes

50 μL of polyplexes were obtained by mixing same volumes of siRNA solution (0.2 mg mL^–1^) and polymer dispersion, obtained from a physical mixture of PHEA-VS-*g*-(PMeOx;bAPAE) and PHEA-VS-*g*-(*pro*PMeOx;bAPAE) (99:1 w/w) to give polymer/siRNA weight ratios (R) equal to 5 and 10. The mixture was mixed gently by pipetting and laved in incubation for 30 min. Subsequently, the obtained polyplexes were incubated with Pen, at a concentration of 0.05 mg mL^–1^, by adding 1.2 and 2.4 μL for R values of 5 and 10, respectively. In addition, 6.4 μL of CuSO_4_ (0.125 mg mL^–1^) and 6 μL of ascorbic acid (0.250 mg mL^–1^) were added as catalysts. After 30 min, HPLC analysis was carried out to quantify the unbound peptide using a Phenomenex Luna C18 column. Chromatographic separation was performed by gradient elution with two solvents: H_2_O containing 0.1% trifluoroacetic acid (solvent A) and ACN containing 0.1% trifluoroacetic acid (solvent B). The initial ratio between the mobile phases A: B was 90:10 and the amount of solvent B was gradually increased until 100% of B in 60 min, at a constant flow rate of 1 mL min^–1^. Detection was carried out at a wavelength of 220 nm, and the peptide exhibited a retention time of 10.6 min. Following analysis, the polyplexes were purified using Vivaspin 20 centrifugal concentrators with a 3 kDa molecular weight cutoff (MWCO), and DLS and zeta potential measurements were performed.

### 4.16 Evaluation of Polyplexes-Mucin Interactions

Interactions between polyplexes and mucin were evaluated by a turbidimetric method. Polyplexes (60 μL; weight ratio 10), prepared with or without the Pen peptide, were combined with an equal volume of mucin solution (2 mg mL^–1^ in 10 mM HEPES, pH 7.4). Samples were incubated at 37 °C and turbidity was followed by recording absorbance at 500 nm every 50 min for up to 6 h using a microplate reader. Background signals from polyplexes in buffer and from mucin alone (1 mg mL^–1^ final concentration) were subtracted. Chitosan served as a positive control, and results were reported as percent transmittance calculated as %*T* = [(*T*_500_ mix – *T*_500_ polyplexes)/*T*_500_ mucin] × 100, where *T* = Abs^–1^.

### 4.17 MTS Cell Viability Assay

Cell viability of 16-HBE cells was assessed by MTS assay (Promega). Cells were seeded in 96-well plates (20,000 cells/well) and, after 24 h, exposed to 200 μL of OPTI-MEM containing PHEA-VS-*g*-(PMeOx;bAPAE) at concentrations from 0.005 to 0.5 mg mL^–1^. All samples were sterilized by 0.22 μm filtration. After 24 or 48 h, cells were washed, incubated with fresh DMEM and MTS reagent, and absorbance was measured at 490 nm. Viability was expressed as a percentage relative to untreated controls. Experiments were performed in triplicate. The same procedure was used to evaluate cytocompatibility of PHEA-VS-*g*-(PMeOx;bAPAE)/siNC polyplexes (polymer/siRNA ratios 5 and 10, ± Pen peptide; 100 nM siRNA) after 24 and 48 h of incubation.

### 4.18 Cell Uptake Study

Cellular uptake of the polyplexes was investigated in 16-HBE cells. For qualitative analysis, cells were seeded on 8-well chambered coverglasses (15,000 cells/well) and, after 24 h, incubated with 200 μL of OPTI-MEM containing polyplexes based on AlexaFluor 488–labeled PHEA-VS-*g*-(PMeOx;bAPAE) and Cy5-labeled siRNA, prepared at polymer/siRNA weight ratios of 5 or 10, with or without Pen peptide (final siRNA concentration: 100 nM). After 24 h, cells were washed, fixed with 4% formaldehyde, stained with DAPI, and imaged by confocal microscopy (Olympus FluoView FV10i). For quantitative uptake, cells were plated in 96-well plates (25,000 cells/well) and treated with 150 μL of the same formulations. After 24 h, cells were washed, lysed (2% SDS, 1% Triton X-100 in DPBS), and fluorescence was measured by spectrofluorometry (AlexaFluor 488:480/520 nm; Cy5:649/670 nm). Fluorescence was normalized to protein content determined by BCA assay. Untreated cells and naked siRNA served as controls. All experiments were performed in triplicate, using sterile-filtered reagents.

### 4.19 Endosomal Escape Study

The endosomal release of polyplexes was investigated in 16HBE cells by using confocal laser scanning microscopy (CLSM). Cells were seeded on 8-well chambered coverglasses (10,000 cells/well) and, after 24 h, incubated with 200 μL of OPTI-MEM containing polyplexes based on PHEA-VS-*g*-(PMeOx;bAPAE) and Cy5-labeled siRNA, prepared at polymer/siRNA weight ratios of 5 or 10, with or without Pen peptide (final siRNA concentration: 100 nM). After 24 h, cells were washed and incubated with DMEM (without phenol red) containing Lysotracker Green (75 nM) for 1 h at 37 °C to label acidic compartments. After PBS washes, nuclear staining was performed using Hoechest for additional 60 min. Excess stains were removed by two final PBS washes. Fluorescence imaging was performed using a Olympus FluoView FV10i Confocal Laser Scanning microscope equipped with a 60× objective. The confocal images were processed using the software ImageJ with the plugin JACoP to assess Pearson’s correlation coefficient.

### 4.20 Gene Silencing Study

MDA-MB-231/LUC cells were seeded into 96-well plates at 1.5 × 10^4^ cells per well in 200 μL of complete medium. Once cells adhered, transfection was performed for 72 h using 150 μL of OPTI-MEM containing PHEA-VS-*g*-(PMeOx;bAPAE)/siGL2/3 or PHEA-VS-*g*-(PMeOx;bAPAE)/siGL2/3-Pen polyplexes, prepared at polymer/siRNA weight ratios of 5 or 10 and providing a final siRNA concentration of 200 nM. Turbofect-complexed siGL2/3 and naked siGL2/3 were used as positive and negative controls, respectively. After treatment, cells were rinsed twice with DPBS and lysed in 50 μL of lysis buffer. Firefly luciferase activity was measured using the Pierce Firefly Luciferase Glow Assay Kit following the manufacturer’s instructions, while total protein levels (25 μL) were quantified by BCA assay. Luciferase activity was normalized to protein content and expressed as a percentage of control. All conditions were tested in triplicate. Samples transfected with scrambled siRNA (siNC) were included, and the same experimental design was repeated to evaluate cell viability.

### 4.21 In Vitro Pulmonary Drug Deposition

The aerosolization of aqueous PHEA-based polyplex dispersions was evaluated in vitro using an Andersen Cascade Impactor (ACI; InPharmaTEC, Italy). The ACI was cooled at 4 °C for 90 min to limit droplet evaporation. Polyplex dispersions (2 mL, including 200 μL of fluorescent polyplexes, PHEA:siRNA = 10) were nebulized for 15 min at 29 L/min using an air-jet nebulizer, with a vacuum flow of 4.5 L/min. The corresponding aerodynamic cutoff diameters for each stage are stage 0 (9 μm), stage 1 (5.8 μm), stage 2 (4.7 μm), stage 3 (3.3 μm), stage 4 (2.1 μm), stage 5 (1.1 μm), stage 6 (0.7 μm), stage 7 (0.4 μm). Deposited material was recovered from each stage with water and quantified by fluorescence (490/515 nm). From cumulative deposition profiles, fine particle fraction (FPF%, < 5.0 μm), mass median aerodynamic diameter (MMAD), and geometric standard deviation (GSD). Experiments were performed in triplicate.

## Supplementary Material



## Notes

^1^H NMR spectra of PMeOx, *pro*PMeOx, PHEA-DV, and PHEA-VS-*g*-(PMeOx;bAPAE); DLS analysis in 10 mM acetate buffer (pH 5.5) at various weight ratios (R); STEM image of PHEA-VS-*g*-(PMeOx;bAPAE)/siRNA polyplexes; siRNA release profile; gene silencing efficiency and cell viability of MDA-MB-231 cells after 72 h of incubation with various formulations without PEN; endosomal localization by CLSM in 16HBE; and fitting function used for the potentiometric titration (PDF)
