# Supplementary material for: Penetratin Decoration Increases RNAi Silencing Effects of Polymeric Carriers
Source: Chem Mater. 2026 Apr 27;38(9):4754–67. doi: 10.1021/acs.chemmater.6c00522 (PMC13177185; doi:10.1021/acs.chemmater.6c00522)
Supplement: Supplementary file 1 [file cm6c00522_si_001.pdf]

Supporting information

Penetratin Decoration Increases RNAi Silencing Effects of Polymeric Carriers

Salvatore Emanuele Drago, Marta Cabibbo, Cinzia Scialabba, Emanuela Fabiola Craparo, Gennara Cavallaro*

Lab of Biocompatible Polymers, Department of Biological, Chemical and Pharmaceutical Sciences and Technologies (STEBICEF), University of Palermo, Via Archirafi 32, Palermo, 90123, Italy

***Correspondence:** emanuela.craparo@unipa.it

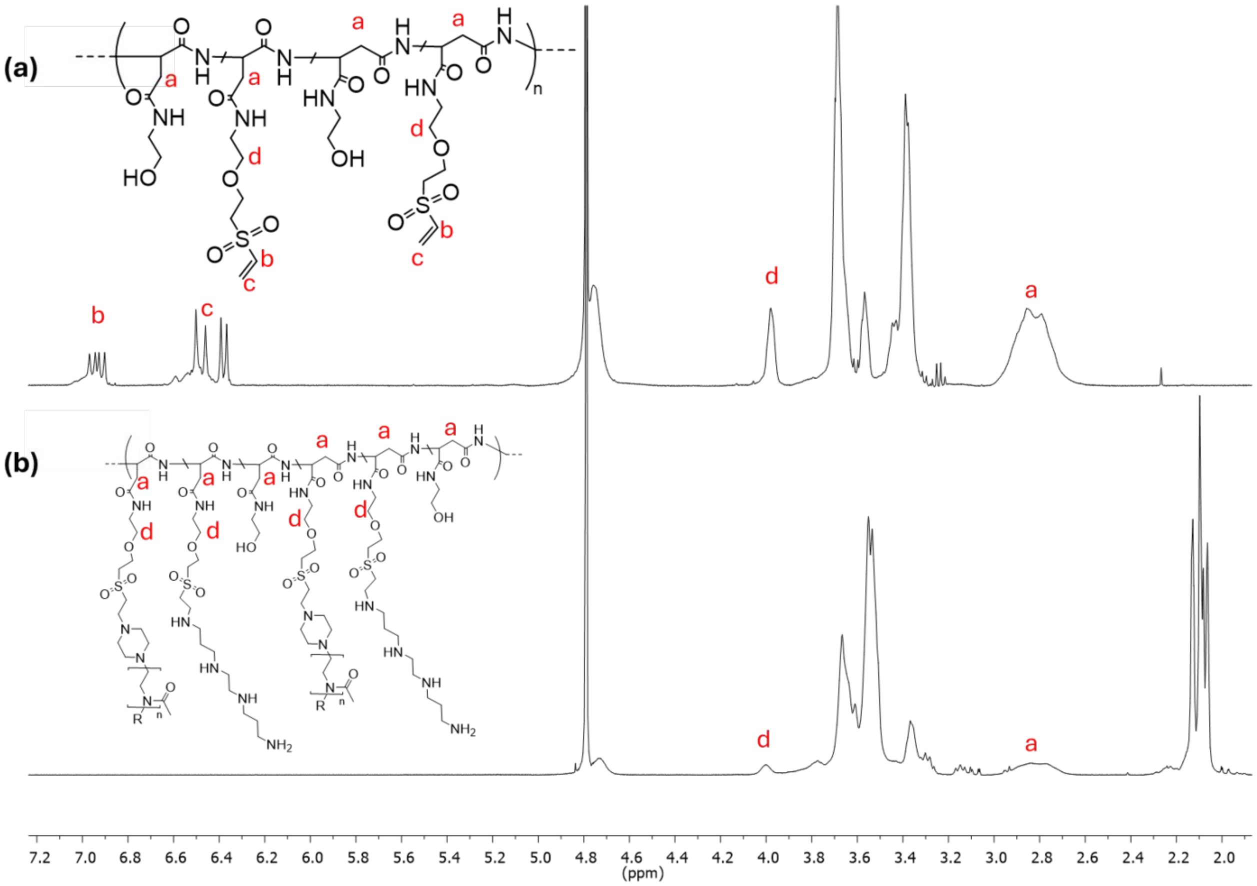

Figure S1. ^{1}H-NMR spectra in D_2O of: (a) PHEA-DV, and (b) PHEA-VS-g-(PMeOx;bAPAE) copolymer (pD ≈3).

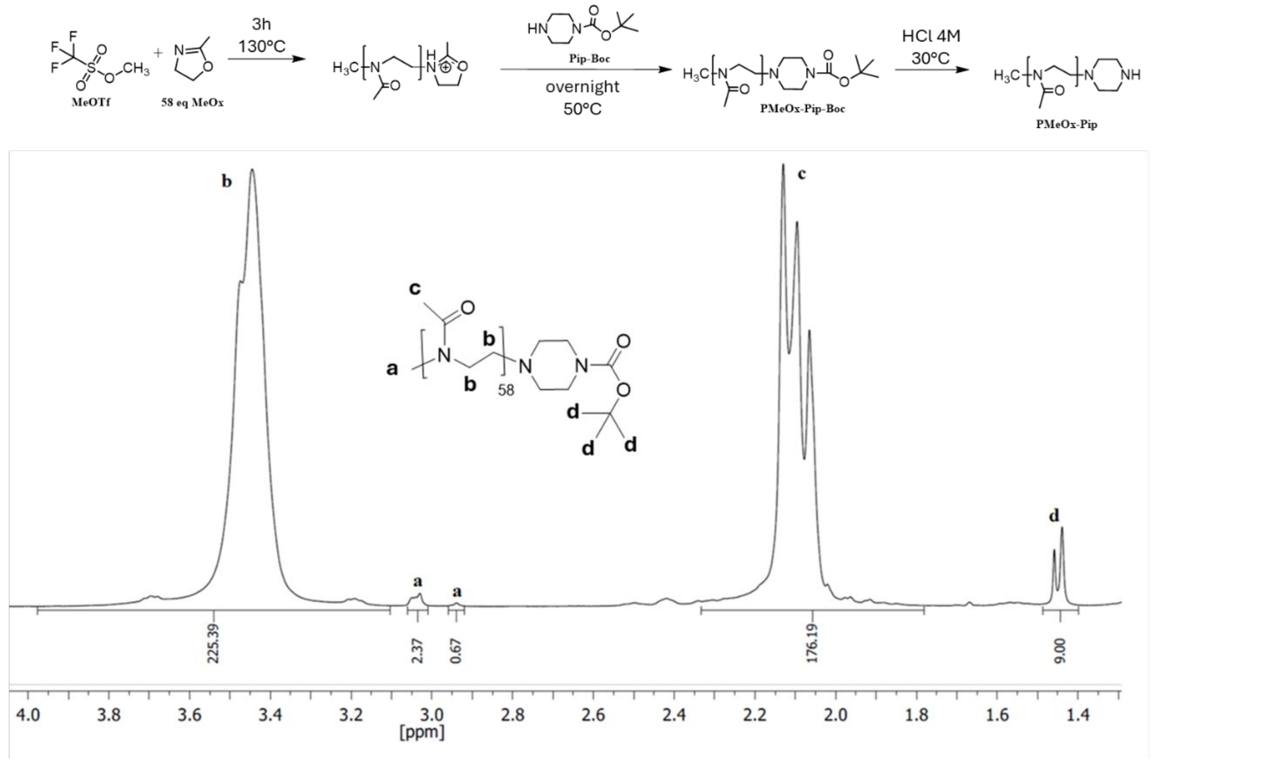

Figure S2. Synthesis and ^{1}H-NMR spectrum of PMeOx in $CDCl_3$.

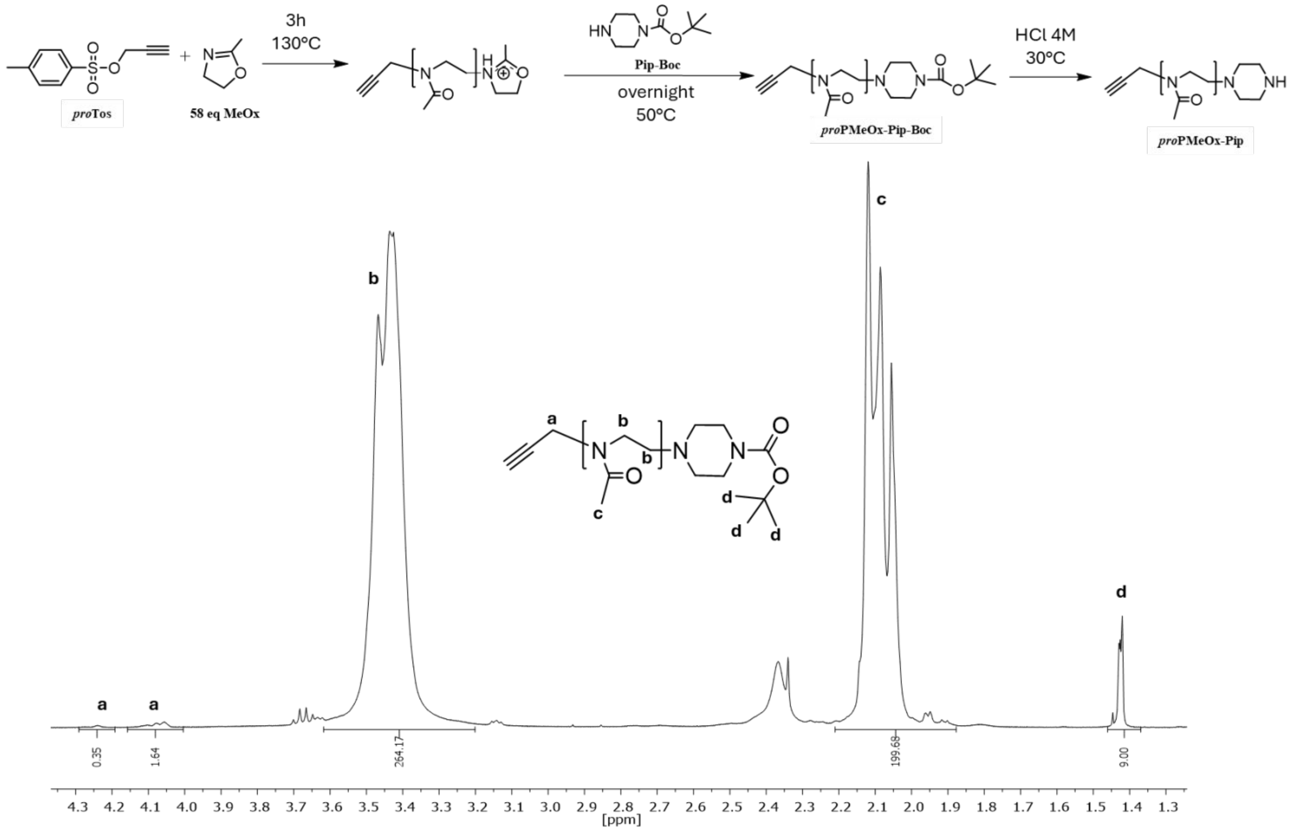

Figure S3. Synthesis and ^{1}H-NMR spectrum of *pro*PMeOx in $CDCl_3$.

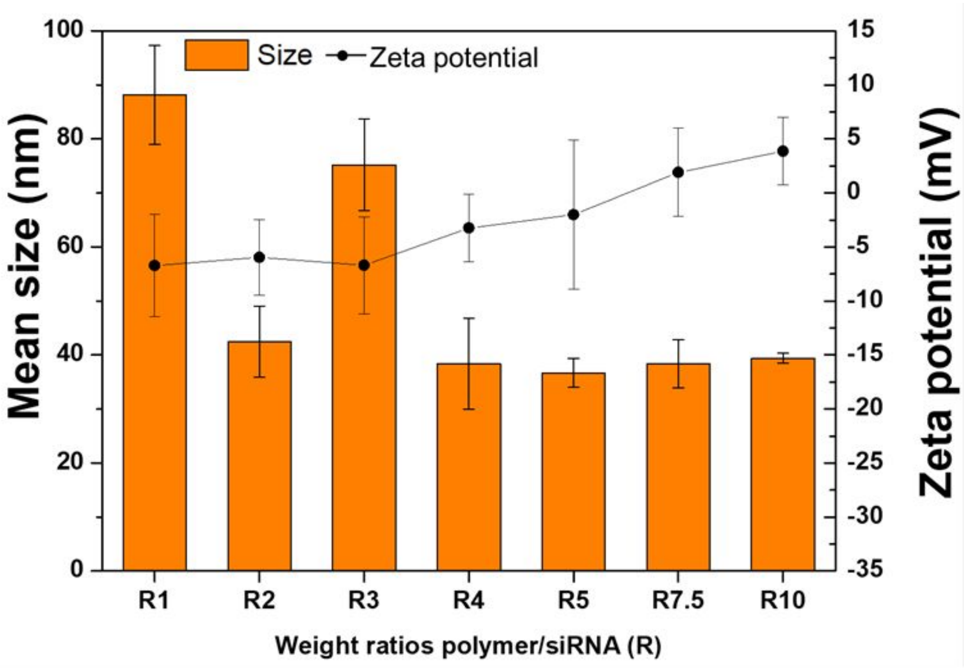

Figure S4. Mean size (istogram) and zeta potential (continued line) of PHEA-VS-g-(PMeOx;bAPAE)/siRNA polyplexes measured by DLS in 10 mM Acetate buffer (pH 5.5) at various weight ratios (R) (data are reported as means ± SD, n = 3).

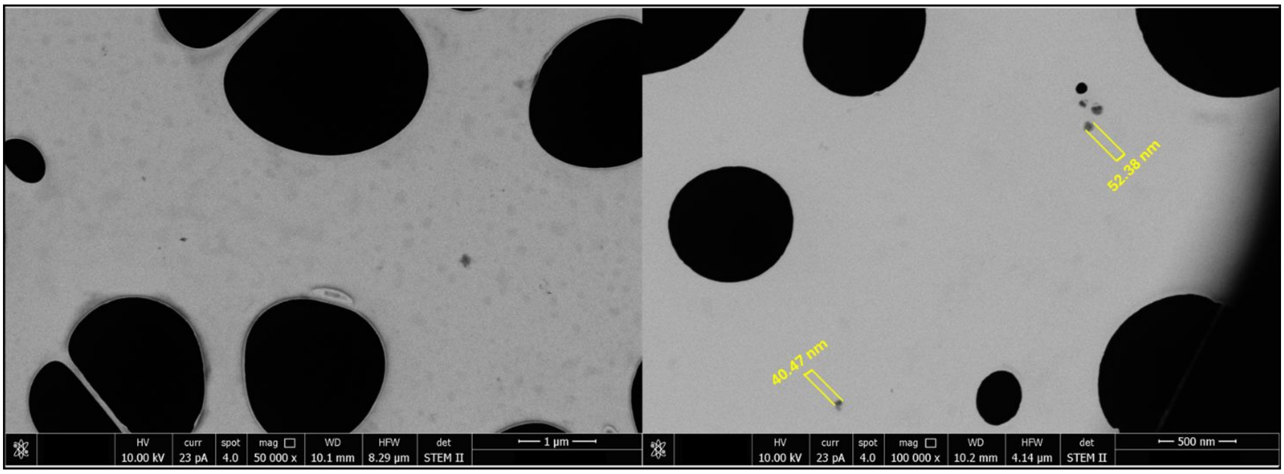

Figure S5. STEM image of PHEA-VS-g-(PMeOx;bAPAE)/siRNA polyplexes.

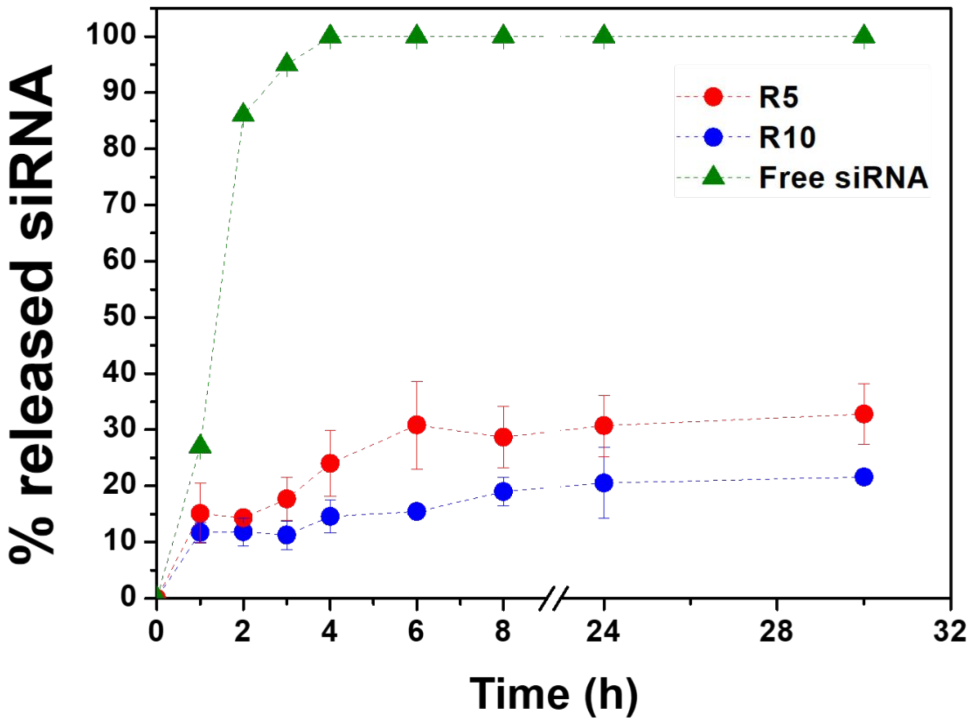

Figure S6. siRNA release from PHEA-VS-g-(PMeOx;bAPAE)/siRNA polyplexes R5 and R10 compared with free siRNA.

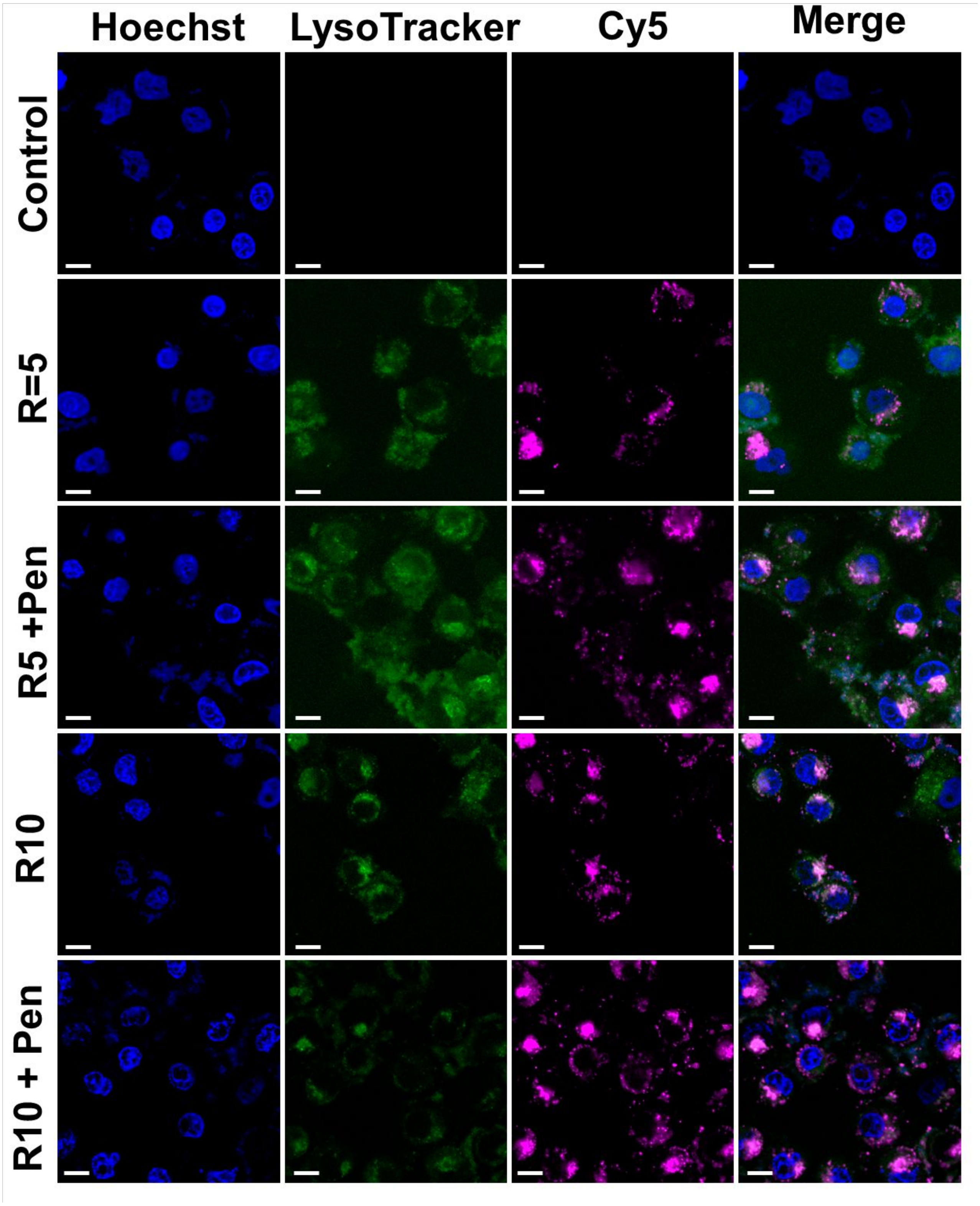

Figure S7. Endosomal localization by CLSM in 16HBE cells after 24h of incubation with samples (100 nM siRNA/well) (bar represents 10 μm).

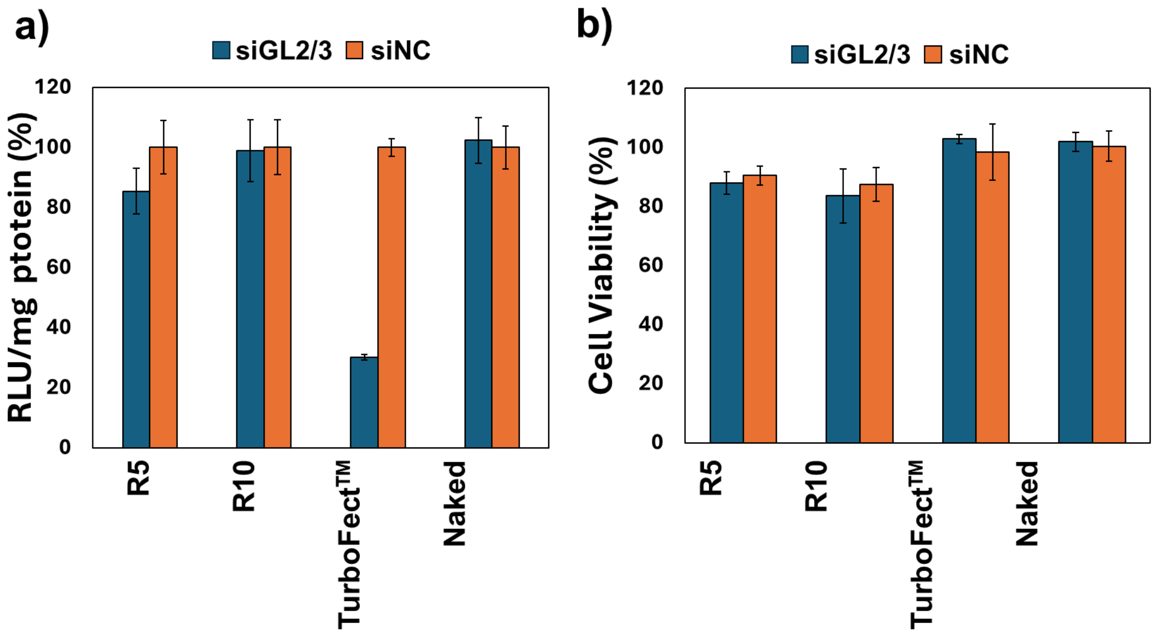

Figure S8. (a) Gene silencing efficiency and (b) cell viability of MDA-MB-231 cells after 72 hours of incubation with various formulations containing either free or complexed siGL2/3 (red) or scrambled siRNA (siNC, blue) at a final concentration of 200 nM siRNA per well. Data are expressed as means ± SD (n = 3).

For the titration, the fitting function obtained is the following:

$$V_B = -\frac{V_0[C_0(4\alpha_4+3\alpha_3+2\alpha_2+\alpha_1)+\Delta]+V_A(\Delta-C_A)}{\Delta+C_B} \quad (1)$$

$$\Delta = C_{H^+} - \frac{K_W}{C_{H^+}\cdot y^2} + C_B \quad (2)$$

$$\log y = -0{,}5\left(\frac{\sqrt{I}}{1+\sqrt{I}} - 0{,}3I\right) \quad (3)$$

where V_A and C_A are the volume and molarity of HCl used for the forward titration, respectively, while V_B and C_B are the volume and molarity of NaOH for the backword titration, respectively, V_0 is the volume of PHEA-VS-g-(PMeOX;bAPAE) sample, C_0 is the equivalent bAPAE molarity, while α_4,α_3,α_2,α_1 are the protonation degree, K_W is the dissociation constant of water, C_{H^+} is the H^+ concentration, y is the activity coefficient, and I is the ionic strength.
